# Spatial Characteristics of Roughness Sublayer Mean Flow and Turbulence Over a Realistic Urban Surface

**DOI:** 10.1007/s10546-016-0157-6

**Published:** 2016-04-22

**Authors:** M. G. Giometto, A. Christen, C. Meneveau, J. Fang, M. Krafczyk, M. B. Parlange

**Affiliations:** 1 School of Architecture, Civil and Environmental Engineering, École Polytechnique Fédérale de Lausanne, Lausanne, Switzerland; 2 Geography/Atmospheric Science Program, University of British Columbia, Vancouver, BC Canada; 3 Mechanical Engineering, Johns Hopkins University, Baltimore, MD USA; 4 Institute for Computational Modeling in Civil Engineering, TU Braunschweig, Braunschweig, Germany; 5 Civil Engineering, Faculty of Applied Sciences, University of British Columbia, Vancouver, BC Canada

**Keywords:** Large-eddy simulation, Turbulence, Turbulent kinetic energy budget, Urban canopy, Urban roughness sublayer

## Abstract

Single-point measurements from towers in cities cannot properly quantify the impact of all terms in the turbulent kinetic energy (TKE) budget and are often not representative of horizontally-averaged quantities over the entire urban domain. A series of large-eddy simulations (LES) is here performed to quantify the relevance of non-measurable terms, and to explore the spatial variability of the flow field over and within an urban geometry in the city of Basel, Switzerland. The domain has been chosen to be centered around a tower where single-point turbulence measurements at six heights are available. Buildings are represented through a discrete-forcing immersed boundary method and are based on detailed real geometries from a surveying dataset. The local model results at the tower location compare well against measurements under near-neutral stability conditions and for the two prevailing wind directions chosen for the analysis. This confirms that LES in conjunction with the immersed boundary condition is a valuable model to study turbulence and dispersion within a real urban roughness sublayer (RSL). The simulations confirm that mean velocity profiles in the RSL are characterized by an inflection point $z_{\gamma }$ located above the average building height $z_\mathrm{h}$. TKE in the RSL is primarily produced above $z_{\gamma }$, and turbulence is transported down into the urban canopy layer. Pressure transport is found to be significant in the very-near-wall regions. Further, spatial variations of time-averaged variables and non-measurable dispersive terms are important in the RSL above a real urban surface and should therefore be considered in future urban canopy parametrization developments.

**Electronic supplementary material:**

The online version of this article (doi:10.1007/s10546-016-0157-6) contains supplementary material, which is available to authorized users.

## Introduction

Accurate modelling of flow and turbulence in the urban roughness sublayer (RSL), the atmospheric layer from the ground to 2–5 times the average building height $z_\mathrm{h}$, is essential to predicting weather, air quality, and the dispersion of gases in the urban environment. Within the RSL, flow and turbulence exhibit strong spatial variations in both the vertical and the horizontal directions, variations that are caused by the flow around the local configuration of roughness elements (buildings and trees). Hence, one-dimensional surface scaling relying on horizontal homogeneity such as the Monin–Obukhov similarity theory (MOST) is not applicable in the RSL (Rotach 1999; Roth 2000). MOST is strictly applicable only in the inertial sublayer (ISL), whose existence in urban environments is subject to debate (Jimenez 2004). Consequently three-dimensional approaches such as computational fluid dynamics (CFD) are required to properly describe flow, turbulence and vertical exchange in the RSL.

However, for many applications, building-resolving information is neither required nor are CFD approaches computationally feasible. In mesoscale weather forecasting and air pollution dispersion models, urban canopy parametrizations (UCP) are used to represent the effects of urban surfaces. UCPs rely usually on a horizontally-averaged approach, where the RSL is represented as a 1D column, often for simplified geometries such as infinite street canyons or cubical blocks of buildings. The vast majority of UCPs use MOST relationships to compute vertical fluxes of momentum and scalars such as heat, humidity and pollutants between the urban facets and the atmosphere, irrespective of the problems outlined above (Grimmond et al. 2010).

Proper techniques to reintroduce a 1D approach in a truly three-dimensional RSL should account for the inherently variable canopy morphology, and its hierarchical structure of scales (from the street or canyon scale to the regional scale) as discussed in Britter and Hanna (2003). For instance, in the horizontal averaging process of the Reynolds-averaged Navier–Stokes (RANS) equations, additional terms arise in the time-averaged momentum balance, called *dispersive fluxes* (Raupach and Shaw 1982), which physically represent spatial correlations between mean vertical flow around buildings and the time-averaged quantity exchanged. The very few modelling studies directly determining dispersive fluxes by means of CFD have shown that these terms can be highly relevant, in addition to Reynolds stress, to the overall momentum transfer in the RSL over rigid canopies (Coceal et al. 2006; Martilli and Santiago 2007).

From a fundamental perspective, efforts using experimental and numerical approaches have been devoted to studying RSL dynamics and scalings over simplified urban-like surfaces, mostly in the form of staggered/aligned cubical arrays (Cheng and Castro 2002; Xie and Castro 2006; Coceal et al. 2006; Cheng and Porté-Agel 2013, 2015; Anderson et al. 2015). The few characteristic length scales that characterize roughness elements in such arrays provide a setting that simplifies simulation, analysis and the development of theory. The approach is justified on the grounds that one should first understand flow over rough surfaces in its simplest form, before introducing complexities such as variable roughness height or shapes, which would result in a broader spectrum of scales and dynamics. However, flow over cubes might be difficult to compare with flow over real urban canopies, where the additional set of length scales, connected to the intrinsic heterogeneity of the surface, might completely modify the dynamics of the system. For instance, boundary-layer flow over surface-mounted cubes with variable element heights, Cheng and Castro (2002) report a thinner ISL when compared with uniform height settings, suggesting an ISL region might not even exist in certain realistic urban canopies. Recent simulations of flow over cubes (Yang et al. 2016) have shown that at high Reynolds numbers, the mean velocity profiles exhibit exponential and logarithmic layers, even for cases with a considerable range of varying cube heights. Further, the effects of building representation and clustering in flows over realistic urban canopies also influence the dynamics of the system (Bou-Zeid et al. 2009).

In the past few decades experimentalists have devoted significant efforts to measuring the relevant processes that drive mean flow and turbulence in the RSL over real cities (Grimmond and Oke 1999; Eliasson et al. 2006; Christen et al. 2007; Ramamurthy et al. 2007; Christen et al. 2009; Peng and Sun 2014; Wang et al. 2014; Ramamurthy and Pardyjak 2015). However, such field studies are limited to measurements at a few points and cannot capture the full three-dimensional flow field in its heterogeneous state. The lack of homogeneity in the statistical properties of the flow within the RSL raise questions on the use of point measurements as a surrogate of horizontally-averaged quantities, as proposed by Rotach (1993a, b) and Christen et al. (2009). The strong spatial variability of the flow represents in fact the main challenge preventing the development of a comprehensive physically-based theory for the vertical structure of the RSL, such as the classic similarity approach (Monin and Obukhov 1954) for the idealized surface layer.

The increased availability of high resolution digital datasets on urban morphology (e.g. high resolution lidar scans, vectorial models based on surveyed data, etc.) encourages the use of real topographies in CFD studies (see for instance Kanda et al. 2013). Further, advances in computational power now allows the representation of the three-dimensional processes of interest at the neighbourhood scale ($\mathcal {O}(10^2-10^3) \ \mathrm {m}$). This is at least allowing constraints to be relaxed with regard to the feasibility and cost of numerical simulations over real urban morphologies.

Output from numerical models, such as large-eddy simulation (LES), can be used to understand the physics of the flow and quantify the most relevant terms and processes that occur in a realistic urban RSL. This is the goal of the current study. Here LES is used to resolve the airflow over and within a detailed urban geometry to, (1) spatially characterize mean flow and turbulence in the RSL, (2) to determine the role of non-measurable terms such as dispersive momentum fluxes, wake production, dispersive transport, pressure transport, dissipation of turbulent kinetic energy (TKE), and (3) to determine how representative are single-point measurements, when used as a surrogate for horizontally-averaged quantities over the entire urban domain. Such information can then be used to guide and validate current upscalings for one-dimensional UCPs.

Throughout the study the Einstein notation is alternated with the vector notation, based on convenience, with *x*, *y*, *z* denoting the streamwise, spanwise and vertical coordinates. The boundary-layer height is denoted as $\delta$ whereas a given height in the domain is $z_\mathrm{label}$, where the subscript “label” refer to various specific heights. Further, $\widetilde{(\cdot )}$ is used to denote a spatially filtered variable (the spatial filtering that is implicitly understood in LES), $\overline{(\cdot )}$ is time-averaging or ensemble averaging (depending on the context), $\langle \cdot \rangle$ is horizontal (*x*, *y*) averaging, time fluctuations are written as $(\cdot )^{\prime }$ (therefore $\overline{(\cdot )^{\prime }} = 0$) and departures of time-averaged terms with respect to their horizontal mean are denoted as $\overline{(\cdot )}^{\prime \prime }$ (therefore $\langle \overline{(\cdot )}^{\prime \prime } \rangle = 0$); $(\cdot )^*$ denotes a normalized variable.

## Materials and Methods

The LES approach is based on the assumption that the energy containing scales of the flow are explicitly resolved. These large-scale motions are the main contributors to the transport of momentum, but due to their strong dependence on boundary conditions and to their intrinsic anisotropy, their effects are difficult to parametrize, typically leading to complex RANS closure models. LES aims instead at providing an adequate model for the “small scales” of the flow, ideally belonging to the inertial subrange of turbulence (Meneveau and Katz 2000), which allows simple parametrizations to be very effective, and it is implicitly assumed that the large-scale motions are properly resolved by the chosen numerical scheme.

### Numerical Algorithm

The isothermal filtered Navier–Stokes equations are solved in their rotational form (Orszag and Pao 1975), to ensure conservation of mass and kinetic energy

1a $$\begin{aligned}&\frac{\partial \tilde{u}_i}{\partial t} + \tilde{u}_j \left( \frac{\partial \tilde{u}_i}{\partial x_j} - \frac{\partial \tilde{u}_j}{\partial x_i}\right) = - \frac{\partial \tilde{\pi }}{\partial x_i} - \frac{\partial \tau _{ij}^{\mathrm{SGS}}}{\partial x_j} - \varPi _1 + \tilde{f}_i^{\varGamma _{\mathrm {b}}} \quad \text {in }\varOmega \times [0,T], \end{aligned}$$

1b $$\begin{aligned}&\frac{\partial \tilde{u}_i}{\partial x_i} =0\;\;\;\;\quad \qquad \qquad \qquad \qquad \qquad \qquad \qquad \qquad \qquad \qquad \qquad \qquad \text {in }\varOmega \times [0,T], \end{aligned}$$

1c $$\begin{aligned}&\frac{\partial \tilde{u}}{\partial z} = \frac{\partial \tilde{v}}{\partial z} = \tilde{w} = 0\;\; \qquad \qquad \qquad \qquad \qquad \qquad \qquad \qquad \qquad \qquad \text {in}\;\varGamma _{\mathrm {top}} \times [0,T], \end{aligned}$$

1d $$\begin{aligned}&(\tilde{\mathbf{u}} \cdot \tilde{\mathbf{n}}) \tilde{\mathbf{n}} = \tilde{\mathbf{u}}_N = 0\qquad \qquad \;\;\; \qquad \qquad \qquad \qquad \qquad \qquad \qquad \qquad \text {in }\varGamma _{\mathrm {b}} \times [0,T], \end{aligned}$$

1e $$\begin{aligned}&\tilde{\mathbf{t}} = - \left( \frac{ \kappa ( \tilde{\mathbf{u}} - \tilde{\mathbf{u}}_N )}{\ln {(1+\varDelta /z_0)}} \right) ^2\;\;\;\; \qquad \qquad \qquad \qquad \qquad \qquad \qquad \qquad \text {in } \varGamma _{\mathrm {b}} \times [0,T]. \end{aligned}$$

Here $\tilde{u}_i$ are the filtered velocity components in the three coordinate directions, $\tilde{\pi }$ is a modified filtered pressure field, namely $\tilde{\pi } = {\tilde{p}}/{\rho } + \frac{1}{3}\tau _{ii}^{\mathrm{SGS}} + \frac{1}{2}\tilde{u}_i\tilde{u}_i$, $\rho$ is a reference density, $\tau _{ij}^{\mathrm{SGS}}$ is the subgrid-scale (SGS) stress tensor (resulting from the filtering operation Pope 2000), $\varPi _1 = \frac{1}{\rho } \frac{\partial \tilde{p}_{\infty }}{\partial x_i}\delta _{i1} < 0$ is a pressure gradient that is introduced to drive the flow, and $\tilde{f}_i^{\varGamma _{\mathrm {b}}}$ is a forcing term that is used to impose the desired boundary condition at the surface location; $\tilde{f}_i^{\varGamma _{\mathrm {b}}}$ has a finite value at the buildings interface $(\varGamma _{\mathrm {b}})$ and is zero elsewhere. Further, $\tilde{\mathbf{t}}$ is the stress vector at the surface location; $\tilde{\mathbf{u}}_N$ is the normal-to-surface velocity vector, $\varDelta = (\mathrm{d}x \times \mathrm{d}y \times \mathrm{d}z)^{1/3}$ and $z_0$ is the hydrodynamic roughness length parameter. The argument of the logarithmic function in Eq. 1 has been regularized by adding a unity constant (Chester et al. 2007).

The LES algorithm has been previously used to study land-atmosphere interaction processes (Albertson and Parlange 1999a, b) and to develop and test linear and non-linear LES SGS models (Meneveau et al. 1996; Porté-Agel et al. 2000; Porté-Agel 2004; Bou-Zeid et al. 2005; Lu and Porte-Agel 2010, 2013).

Equations are solved in strong form on a regular domain $\varOmega$, a pseudo-spectral collocation approach (Orszag 1969, 1970) based on truncated Fourier expansions is used in the *x*, *y* coordinate directions, whereas a second-order accurate centered finite differences scheme is adopted in the vertical direction, requiring a staggered grid approach for the $\tilde{u},\tilde{v},\tilde{p}$ state variables (these are stored at $(j+1/2)\mathrm{d}z$, with $j=1,nz$). Time integration is performed adopting a fully explicit second-order accurate Adams-Bashforth scheme and a fractional step method (Chorin 1968; Kim and Moin 1985) is adopted to compute the pressure field, which is based on an operator-splitting technique. In addition, non-linear terms are deliased via the 3 / 2 rule (Canuto et al. 2006), to avoid the piling up of energy in the high wavenumber range (Kravchenko and Moin 1997). The computational boundary is partitioned as $\partial \varOmega = \varGamma _{\mathrm {b}} \cup \varGamma _{\mathrm {top}} \cup \varGamma _\mathrm{lateral}$, where $\varGamma _{\mathrm {top}}$ and $\varGamma _\mathrm{lateral}$ denote the top and lateral boundaries respectively. A free-lid boundary condition applies at $\varGamma _{\mathrm {top}}$ and a parametrized boundary condition is prescribed at $\varGamma _{\mathrm {b}}$ (see in Eq. 1). Periodic boundary conditions apply at $\varGamma _\mathrm{lateral}$ due to the Fourier spatial representation.

#### Subgrid-Scale Closure Model

The proposed study considers two LES closure models to evaluate $\tau _{ij}^{\mathrm{SGS}}$: the classical static Smagorinsky model (Smagorinsky 1963) in conjunction with a wall damping function (SMAG), similar to that adopted in Mason and Thomson (1992), and the scale-dependent model with Lagrangian averaging of the coefficient (LASD), developed in Bou-Zeid et al. (2005).

Smagorinsky models rely on the viscous analogy and on the mixing length concept, and evaluate the SGS terms as a function of the resolved strain rate tensor,

2 $$\begin{aligned} \tau _{ij}^{\mathrm{SGS}} = - 2 \nu _{t} \tilde{S}_{ij} = -2 (c_{\mathrm{s},\varDelta } \varDelta )^2 \Vert \tilde{S} \Vert _2 \tilde{S}_{ij}, \end{aligned}$$

where $\nu _{t}$ represents the eddy viscosity, $\varDelta$ is the filter width (usually proportional to the grid size), $\tilde{S}_{ij}$ is the filtered shear rate tensor and $c_{\mathrm{s},\varDelta }$ is the Smagorinsky coefficient at scale $\varDelta$. The two models essentially differ in the way they compute the Smagorinsky coefficient.

The SMAG model prescribes a constant coefficient, whose value is usually that derived from the theory of homogeneous turbulence $(c_{\mathrm{s},\varDelta }=0.16$, for the sharp spectral cut-off filter). However, in applications involving high Reynolds number boundary-layer flows, such as that proposed herein, the model is known to be over-dissipative in the near wall regions, where $c_{\mathrm{s},\varDelta }$ should approach zero. To cope with this we introduce an empirical wall damping function (Mason and Thomson 1992), which has the drawback of requiring an ad hoc calibration for each specific flow case, but partially ameliorates the dissipative properties of the SMAG model.

The LASD model overcomes the necessity of ad hoc specification of the damping function by exploiting the smallest resolved scales to compute the model coefficient at runtime. It represents an evolution of the original dynamic model, based on the Germano identity (Germano et al. 1991) and its modifications (Lilly 1992). LASD relaxes the scale invariance assumption of the model coefficient, which is a desirable property in the near wall regions, where the grid size approaches the limits of the inertial subrange (Meneveau and Katz 2000). The Lagrangian averaging of the model coefficient makes the model well suited for applications involving complex geometries, since it preserves local variability while satisfying Galileian invariance, and overcomes the requirement of homogeneous directions (Bou-Zeid et al. 2005). Additionally, the energy cascade process is more apparent along fluid pathlines (Meneveau and Lund 1994), which enforces the theoretical basis of the model. To reduce the strong Gibbs oscillations that would arise at the interface if adopting a classic spectral cut-off filter, a Gaussian filter is introduced in conjunction with the LASD model, which has the desirable property of being of compact support in both physical and wavenumber space (Tseng et al. 2006).

#### Discrete Forcing Immersed Boundary Method

To model the urban canopy a discrete forcing approach immersed boundary method is adopted (Mohd-Yusof 1997; Mittal and Iaccarino 2005). The buildings’ interface $\varGamma _{\mathrm{b}}(x,y)$ is represented implicitly as the zero level-set of a (higher dimensional) signed distance function $\tilde{\phi }(x,y,z)$, and the computational domain $\varOmega$ is split into two regions: the inside building region $\varOmega _\mathrm{b}$, where $\tilde{\phi } \le 0$, and the fluid region $\varOmega _\mathrm{f}$, where $\tilde{\phi } > 0$. The $\tilde{\phi }(x,y,z)$ function is initialized adopting an iterative projection technique on the triangulated (urban) surface, which has been specifically developed for the current study. The immersed boundary algorithm is a minor modification of the one proposed in Chester et al. (2007). The velocity field is fixed to zero in $\varOmega _\mathrm{b}$ through a penalty method and the law-of-the-wall is enforced at all the collocation nodes that fall in the region $-1.1 \varDelta \le \tilde{\phi } \le 1.1 \varDelta$. The law-of-the-wall is based on the equilibrium logarithmic assumption (Moeng 1984) and computes the local surface stress vector as

3 $$\begin{aligned} \tilde{\mathbf{t}} = - \left( \frac{ \kappa ( \tilde{\mathbf{u}} - \tilde{\mathbf{u}}_N )}{\ln {(1+\varDelta /z_0)}} \right) ^2. \end{aligned}$$

The main difficulty in coupling the immersed boundary method with a pseudo-spectral algorithm is represented by the fact that the domain is not simply connected. The solutions to Eq. 1, in a given plane cutting the building elements, is of class $C^0$, with the discontinuities in first derivatives localized at the building-atmosphere interface $\varGamma _{\mathrm{b}}$. The spectral representation results in Gibbs oscillations in the near interface regions, which will then propagate away from the singularity and degrade the quality of the partial sum approximation (Greer and Banerjee 1997). To alleviate such phenomena a smooth velocity profile $\tilde{u}_i$ is generated in $\varOmega _\mathrm{b}$ ($\tilde{\phi } \le 0$) before the spectral differentiation step (Tseng et al. 2006), adopting a Laplacian smoothing operator that resembles the reconstruction scheme proposed in Cai et al. (1989) and Greer and Banerjee (1997). Alternative smoothing algorithms are also available, as in Fang et al. (2011) and Li et al. (2016).

### Site Description and Instrumentation

Numerical solutions are compared to field data from the Basel Urban Boundary Layer Experiment (BUBBLE), a multi-institutional effort dedicated to the energetics and dispersion processes in the urban boundary layer (Rotach et al. 2005).

**Fig. 1:**
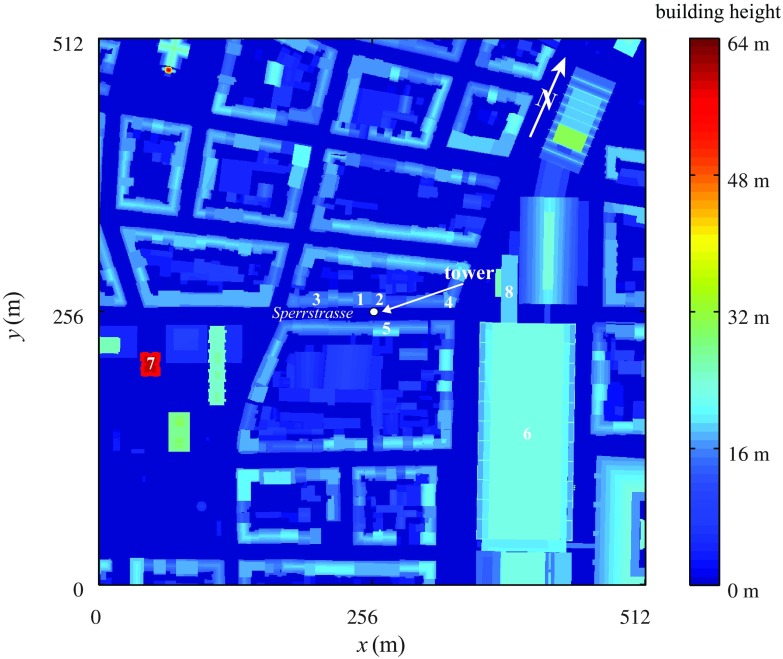
*Colour contour* of the surface height $(\varGamma _{\mathrm {b}}(x,y))$ for a neighbourhood scale of $512\times 512 \ \mathrm {m}$, centered at the tower location. The “Sperrstrasse” street canyon is aligned with the *x* coordinate axis

During BUBBLE, a 32-m high tower was deployed inside the 13-m wide “Sperrstrasse” street canyon in Basel, Switzerland $(47^{\circ }33^{\prime }57.20^{\prime \prime }\mathrm {N}, 7^{\circ }35^{\prime }48.80^{\prime \prime }\mathrm {E}, \mathrm {WGS}\ 84)$, as displayed in Fig. 1. The orientation of the street canyon is along the axis $066^{\circ }$–$246^{\circ }$ (east-north-east to west-south-west), the block where the tower was operated is characterized by a length of $160 \ \mathrm {m}$, and an average width-to-height ratio of $\xi _\mathrm{c}/z_\mathrm{h} = 1.0$, where $\xi _\mathrm{c}$ is the street canyon width and where $z_\mathrm{h}$ is the mean building height. The tower was placed at the midpoint of the block, $3 \ \mathrm {m}$ away from the north wall, and equipped with six ultrasonic anemometer-thermometers (sonics, labels $A-F$ in Table 1), mounted on horizontal booms reaching from the tower into the centre of the street canyon.

Buildings on both sides of the street canyon “Sperrstrasse” have pitched roofs except two flat-roof buildings directly adjacent to the tower on the northern side (labels 1 and 2 in Fig. 1) and two flat-roof buildings close to the two intersections (labels 3 and 4). The height of the buildings typically reaches $15 \ \mathrm {m}$ on both sides; a high pitched roof of $20 \ \mathrm {m}$ is located directly to the south-east of the tower (label 5) (Christen et al. 2009). Sectors from west to north-north-east and south-south-east to south-south-west are similar to structures found immediately around the tower. These sectors are homogeneous in terms of integral morphometric statistics and building height with fetch extending to $700 \ \mathrm {m}$. In the sector north-east to south-south-east an extensive commercial area is found at $100 \ \mathrm {m}$ distance to the tower with flat roofs and roof heights from 20 to 25 m (label 6), whereas an isolated high-rise building of 64 m in height is located ${\approx }200 \ \mathrm {m}$ to the south-west of the tower (label 7). A 18.5-m high building is located approximately $100 \ \mathrm {m}$ north-east of the tower (label 8). For the considered neighbourhood, trees are all of the same height and lower than buildings, and the plan area fraction of vegetation (grass plus trees) is only 0.16 (Christen and Vogt 2004).

**Table 1:** Details on the ultrasonic anemometer-thermometer (sonic) instrumentation, label, absolute measurement heights *z*, normalized measurement heights (the normalization scale is the location of the highest sonic), sonic type, sampling frequency $f \ (\mathrm {Hz})$

Label	$z \ (\mathrm {m})$	$z/z_\mathrm{t}$	Instrument type	$f \ (\mathrm {hz})$
*A*	3.6	0.11	Gill R2 Omnidirectional	20.8
*B*	11.3	0.35	Gill R2 Omnidirectional	20.8
*C*	14.7	0.46	Gill R2 Omnidirectional	20.8
*D*	17.9	0.56	Gill R2 Omnidirectional	20.8
*E*	22.4	0.7	Gill R2 Asymmetric	20.8
*F*	31.7	1	Gill HS	20.0

### The Urban Canopy Dataset

**Fig. 2:**
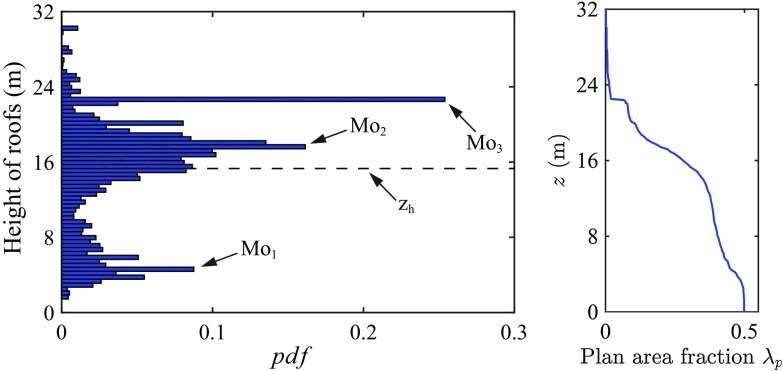
Binned *p.d.f.* of the roof heights (*left*) and plan area fraction $\lambda _\mathrm{p}(z)$ (*right*) for the considered surface ($512 \hbox { m} \times 512 \hbox { m}$)

A high resolution three-dimensional terrain and building digital model (vector format) that includes downtown and sub-urban areas of Basel, was provided by the authorities of the city (GVA Grundbuch und Vermessungsamt Basel-Stadt). The building model includes details such as openings and chimneys, but does not include vegetation; neglecting vegetation is justified considering its small plan area fraction (0.16). The dataset was rasterized at a horizontal resolution of $0.5 \ \mathrm {m}$ and rotated by $-24^{\circ } (\text {clockwise})$ in order to have the main street canyon aligned with the coordinate system (*x*, *y*, *z*), as in Fig. 1. The probability density function (*p.d.f.*) of roof heights is characterized by a trimodal distribution (see left plot in Fig. 2) with modes at $z \approx 4.5 \ \mathrm {m}$ (Mo$_1$), $z \approx 17.5 \ \mathrm {m}$ (Mo$_2$) and $z \approx 22.5 \ \mathrm {m}$ (Mo$_3$). The mean roof height $z_\mathrm{h}$ is $15.3 \ \mathrm {m}$ and the variance of the roof height is $6.4 \ \mathrm {m}$. The first mode Mo$_1$ corresponds to one-storey buildings in the backyards (garages, commercial buildings, etc.), the second mode Mo$_2$ is related to the the main residential (attached) buildings that line streets and enclose courtyards, whereas the third mode Mo$_3$ is linked to building N.6 in Fig. 1, whose large surface has a significant impact on the *p*.*d*.*f* of the surface heights.

### Processing of the Profile Tower Dataset

Velocity components *u*, *v*, *w* and virtual acoustic temperature $\theta$ were continuously recorded at all six levels simultaneously from December 1 2001 to July 15 2002. Data acquisition systems and quality control procedures including wind-tunnel calibrations of the instruments are described and documented in Christen (2005); *u*, *v* and *w* statistical moments up to order three were calculated and stored for blocks over 5 min. No filtering was applied to the signal nor standard de-trending, to ensure energy conservation and enable vertical gradients of the state variables to be properly computed. To provide data for comparison with pressure-driven simulations the following processing is further performed:

1. Data are averaged in blocks of 30 min.
2. Data are selectively sampled from the year-round dataset based on the wind direction computed at the tower top sensor. Only 30-min blocks characterized by an approaching wind direction of $\alpha = 66^{\circ } \pm 10^{\circ }$ (along-canyon regime) and of $\alpha = 156^{\circ } \pm 10^{\circ }$ (across-canyon regime) throughout the $6 \times 5$-min intervals are kept.
3. In order to eliminate the influence of thermal stability, the periods are further filtered based on the classic stability parameter $\zeta = (z - z_\mathrm{d})/L$ (Stull 1988), where *L* is the Obukhov length $(L = \theta u^2_{\tau } / [\kappa g \theta _*])$ calculated with both friction velocity $u_{\tau }$ and scaling temperature $\theta _*$ measured at the tower top. Only periods characterized by near-neutral stability are retained, $-0.1 \le \zeta \le +0.1$. The displacement height is computed as $z_\mathrm{d}=(2/3) z_\mathrm{h}$, in the typical range suggested for high-density urban roughness elements (Grimmond and Oke 1999).
4. Cases characterized by $u_{\tau } \le 0.15 \ \mathrm {m\, s^{-1}}$ at tower top are excluded from the analysis.

Despite the strict constraints, the availability of a relatively long dataset resulted in 30 blocks for the east-north-east wind-approaching direction ($\alpha = 66^{\circ } \pm 10^{\circ }$) and three blocks for the south-south-east wind-approaching direction ($\alpha = 156^{\circ } \pm 10^{\circ }$).

**Table 2:** Geometrical and numerical parameters for the LES runs

ID	$z_0 \ \mathrm {(m)}$	$\alpha$ ($^\circ$)	SGS model
*A*	$\varDelta /15$	66	SMAG
*B*	$\varDelta /30$	66	SMAG
*C*	$\varDelta /15$	156	SMAG
*D*	$\varDelta /30$	156	SMAG
*E*	$\varDelta /15$	66	LASD
*F*	$\varDelta /30$	66	LASD
*G*	$\varDelta /15$	156	LASD
*H*	$\varDelta /30$	156	LASD

### Set-up of Simulations

Simulations are performed over a regular domain, of size $L_x \times L_y \times L_z = 512 \times 512 \times 160$, (horizontally) centered at the tower locations $(x_\mathrm{t},y_\mathrm{t})$ and discretized with a 1-m stencil in the three coordinate directions (*x*, *y*, *z*). Numerical parameters for each run are summarized in Table 2. Two directions of the incoming flow are considered, $\alpha = 66^{\circ }$ and $\alpha = 156^{\circ }$, which correspond to an along-canyon and across-canyon wind regime respectively. The flow is forced by imposing a constant pressure gradient $\partial _x p_{\infty } /\rho$, which, in conjunction with lateral periodic boundary conditions, defines a friction velocity $u_{\tau } = \sqrt{(\delta -z_\mathrm{d})\partial _x p_{\infty }/\rho } \approx 1.23 \text { m s}^{-1}$, making the system independent of Reynolds number effects (fully rough flow regime). Under such conditions it is possible to scale the solution throughout the boundary layer with a characteristic velocity *U*, since molecular diffusion is negligible. The relatively homogeneous integral morphometric statistics and building height in the neighbourhood justifies the pressure forcing in conjunction with lateral periodic boundary conditions (the main surface transition occurs at ${\approx }700 \ \mathrm {m}$ in the radial direction from the tower location). Domain size was chosen based on a sensitivity study (not shown). The hydrodynamic roughness length $z_0$, defining the surface roughness, is not known a priori; here, $z_0$ is defined based on a Nyquist-Shannon representation criterion (Shannon 1949): adopting a reference grid stencil $\varDelta$, the smallest flow/surface feature that can be represented through the Fourier partial sums is $k_{\varDelta } = 2\varDelta$, whereas all scales smaller than $k_{\varDelta }$ need to be modelled. Since $z_0 = 0.033 k_\mathrm{s}$, where $k_\mathrm{s}$ is the equivalent Nikuradse sand grain roughness, and given that $k_\mathrm{s} \rightarrow k$ in the limit of negligible viscous effects (*k* is the height of the considered roughness element), we have that $z_0 = 0.033 k_{\varDelta } \approx \varDelta /15$. To account for variations in the solution due to the $z_0$ parameter, $z_0= \varDelta / 30$ is also considered. To reduce the computational time required to reach a dynamic equilibrium, the initial velocity field for each simulation is imposed through interpolation from results of a run at coarser resolution (twice as coarse in each coordinate direction). Equations are integrated in time for 480 non-dimensional time units $T = z_\mathrm{h}/u_{\tau }$ (${\approx }2 \ \mathrm {h}$ in dimensional time) in the coarser grid, before being used as the initial condition for the finer grid, where they are further integrated for 250*T*. A time 100*T* is required in order to achieve statistical stationarity in the velocity field and 150*T* is used to compute statistics, which ensures convergence of first- and second-order moments to the corresponding expected values. To further reduce computational costs the $\delta /z_\mathrm{h} \gtrapprox 50$ requirement (Jimenez 2004) is here sacrificed; simulations are characterized by $\delta /z_\mathrm{h} = 10.6$. Roughness has a great influence on turbulence up to ${z/z_\mathrm{h} \approx \min (1+D/z_\mathrm{h},5)}$, where *D* is the separation distance between nearest-neighbour roughness elements (Raupach and Thom 1981; Jimenez 2004). Assuming the top of the RSL to be located at $z/z_\mathrm{h}=5$ implies that the current geometry does not allow an ISL to survive. The limited $\delta /z_\mathrm{h}$ in the proposed study might be justified by considering that the focus is on the dynamics within the RSL. In these regions turbulence is expected to be strongly affected by the morphology of the roughness elements and only in a minor part by the dynamics of the logarithmic and outer layers (Anderson 2016).

## Results and Discussion

### Properties of the Instantaneous Velocity Field

**Fig. 3:**
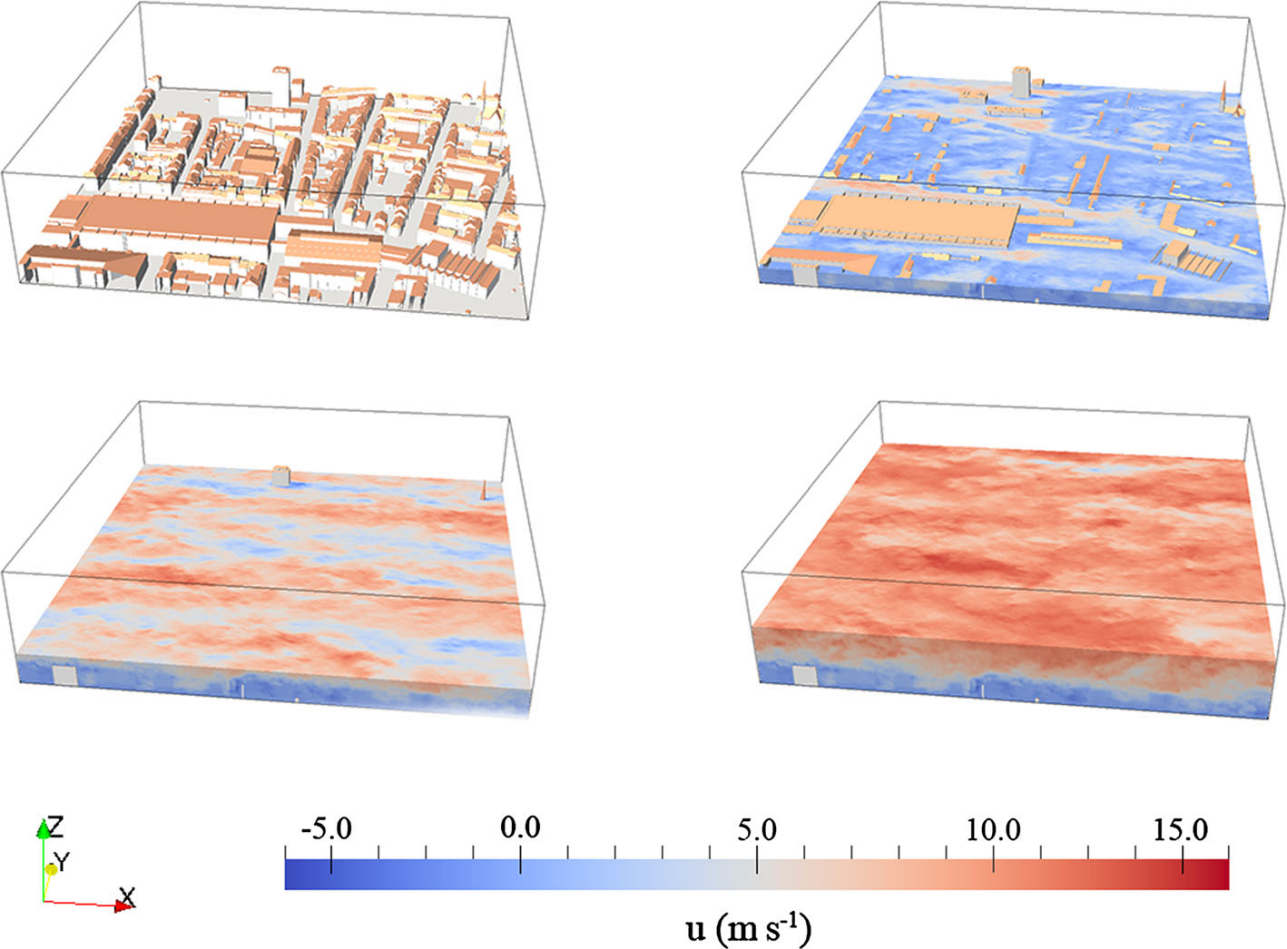
From *top-left* to *bottom-right*: urban canopy, *colour contour* of (dimensional) stream-wise velocity at the planes $z/z_\mathrm{h}=1$, $z/z_\mathrm{h} = 2$, $z/z_\mathrm{h}=4$ for simulation *C* (across-canyon wind direction). $z_\mathrm{h}$ is the average height of buildings in the considered canopy model. The snapshot represents the flow field at $T^* = 250$ (statistically steady state flow regime). Note that the surface model has been rotated so that the street canyon is perpendicular to the *x* axis. An animated version of this figure can be found in the web supplementary material to this article

To provide a qualitative idea of the instantaneous resolved velocity field, a colour contour of the streamwise velocity field from simulation C (across-canyon regime) is displayed in Fig. 3. The flow in the RSL is characterized by a broad spectrum of explicitly resolved length scales, which are heterogeneous in space and strongly depend on the current configuration of buildings.

The relatively high variance characterizing the distribution of roof heights ($\sigma _{z_\mathrm{h}}/z_\mathrm{h} = 0.42$) causes a transitional behaviour between skimming flow and wake interference flow (see definition of flow regimes in Oke 1988), despite the high value of the plan-area fraction covered by buildings (see Fig. 2). The lower part of the RSL ($z / z_\mathrm{h} < 2$) is mainly composed of wake and of non-wake regions (Böhm et al. 2013), whereas higher up in the boundary layer the flow organizes itself into a set of relatively high-speed and low-speed streamwise elongated streaks.

### Mean Flow Velocity

In the following, the double averaging (DA) approach is used to describe the flow field. The DA methodology was initially developed to characterize the flow field over vegetation canopies (Wilson and Shaw 1977; Raupach and Shaw 1982; Finnigan et al. 1984) and has been recently extended to study flows over gravel beds (Nikora et al. 2001, 2007) and flows over rigid canopies (Raupach et al. 1991; Coceal et al. 2006). In the DA framework a general variable $\theta (x,y,z,t)$ is decomposed into a time-space average $\langle \overline{\theta } \rangle (z)$ (bar and brackets denote temporal and spatial averages, respectively), a fluctuation of the time-averaged quantity with respect to its time-space value $\overline{\theta }^{\prime \prime }(x,y,z)$ and a turbulent fluctuation $\theta ^{\prime }$,

4 $$\begin{aligned} \theta (x,y,z,t) = \langle \overline{\theta } \rangle (z) + \overline{\theta }^{\prime \prime }(x,y,z) + \theta ^{\prime }(x,y,z,t). \end{aligned}$$

We here consider the intrinsic averaging approach (Nikora et al. 2007), where averaging is performed over horizontal planes in the fluid domain only, i.e. only the outdoor air, excluding the air volume within buildings, as opposed to the superficial spatial averaging $\langle \overline{(\cdot )} \rangle _\mathrm{s}$ where averaging is performed over the whole horizontal plane (*x*, *y*), including the interior of the roughness elements.

To facilitate comparison with the previous literature, numerical profiles are normalized adopting $u_{\tau } = \sqrt{(\delta -z_\mathrm{d})\partial _x p_{\infty }/\rho }$, whereas measured profiles are first rescaled with the ratio between measured and simulated friction velocities at the tower top location $u_{\tau }(x_\mathrm{t},y_\mathrm{t},z_\mathrm{t})/u_{\tau , \mathrm{tower}}(z_\mathrm{t})$, and then normalized with $u_{\tau } = \sqrt{(\delta -z_\mathrm{d})\partial _x p_{\infty }/\rho }$, i.e.

5 $$\begin{aligned} u_{\tau ,\mathrm{tower}}^*(z) = \frac{u_{\tau }(x_\mathrm{t},y_\mathrm{t},z_\mathrm{t})}{u_{\tau , \mathrm{tower}}(z_\mathrm{t})} \frac{u_{\tau ,\mathrm{tower}}(z)}{u_{\tau }}. \end{aligned}$$

The rescaling of measured profiles ensures that the measured friction velocity at the tower top location matches its numerical (local) counterpart. Simulated and measured length scales are normalized with the mean building height of the entire $512 \times 512$ m domain $(z_\mathrm{h} = 15.3 \ \mathrm {m})$. Throughout error bars in tower measurements denote the standard deviation of sample means, where each sample mean corresponds to a 30-min time average of the considered variable at each $z_i^{\mathrm{tower}}$ height (recall the 30-min average blocks are selected by enforcing the constraints defined in Sect. 2.4). Shaded regions in the numerical profiles are used to denote the standard deviation of a selected variable, at each vertical layer $z_i^\mathrm{LES}$, across the considered SGS models (SMAG and LASD) and hydrodynamic roughness lengths $z_0$. Note that the availability of only three blocks of data for the $\alpha = 156^{\circ }$ approaching wind direction questions the representativeness of the corresponding standard deviations, which might not be good estimates of the population standard deviation.

**Fig. 4:**
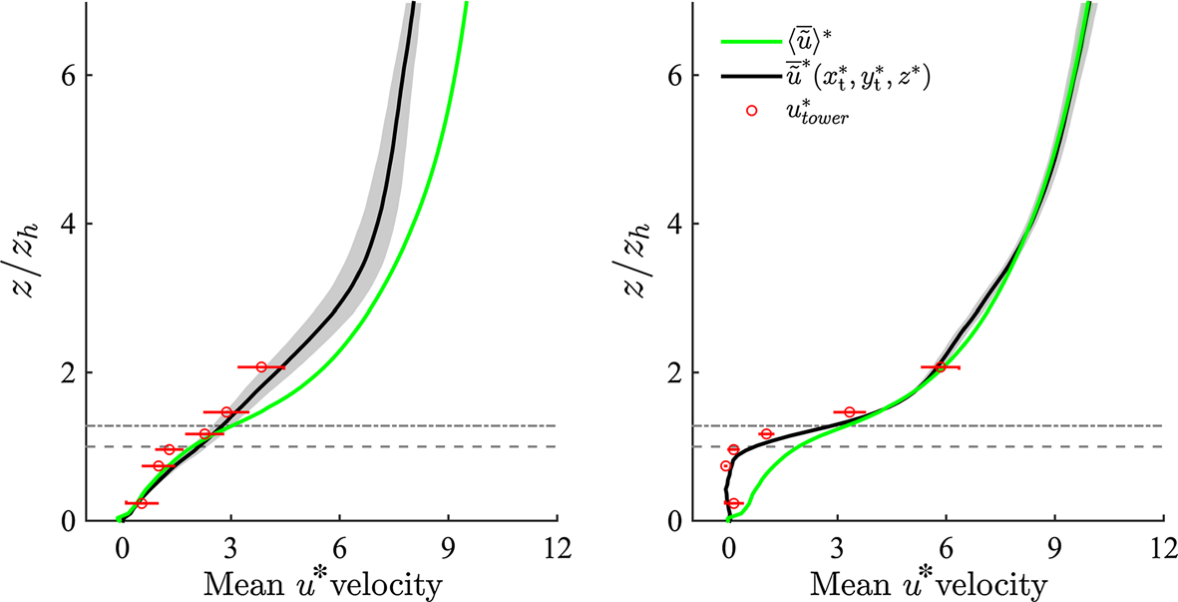
DA LES velocity component $\langle \overline{\tilde{u}}^* \rangle$ (*green*) and comparison between time-averaged LES velocity sampled at the tower location $\overline{\tilde{u}}^*(x_\mathrm{t}^*,y_\mathrm{t}^*,z^*)$ (*black*) and time-averaged tower-measured velocity component $\overline{u}^*_{\mathrm{tower}}$ (*red dots*), for along-canyon wind regime (*left*) and across-canyon wind regime (*right*). *Horizontal dashed* and *dot-dashed* (*grey*) *lines* denote $z_\mathrm{h}$ and $z_{\gamma }$ respectively. Only the lower $75\,\%$ of the domain is shown

**Fig. 5:**
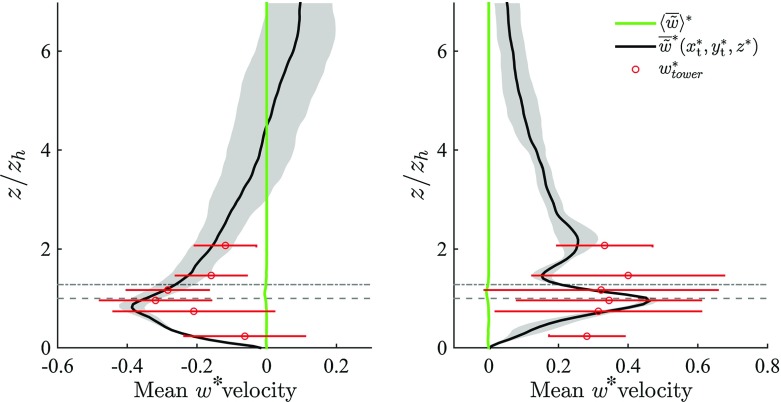
DA vertical LES velocity component $\langle \overline{\tilde{w}}^* \rangle$ (*green*) and comparison between time-averaged LES vertical velocity sampled at the tower location $\overline{\tilde{w}}^*(x_\mathrm{t}^*,y_\mathrm{t}^*,z^*)$ (*black*) and time-averaged tower-measured velocity component $\overline{w}^*_{\mathrm{tower}}$ (*red dots*), for along-canyon wind regime (*left*) and across-canyon wind regime (*right*). *Horizontal dashed* and *dot-dashed* (*grey*) *lines* denote $z_\mathrm{h}$ and $z_{\gamma }$ respectively. Only the lower $75\,\%$ of the domain is shown

**Fig. 6:**
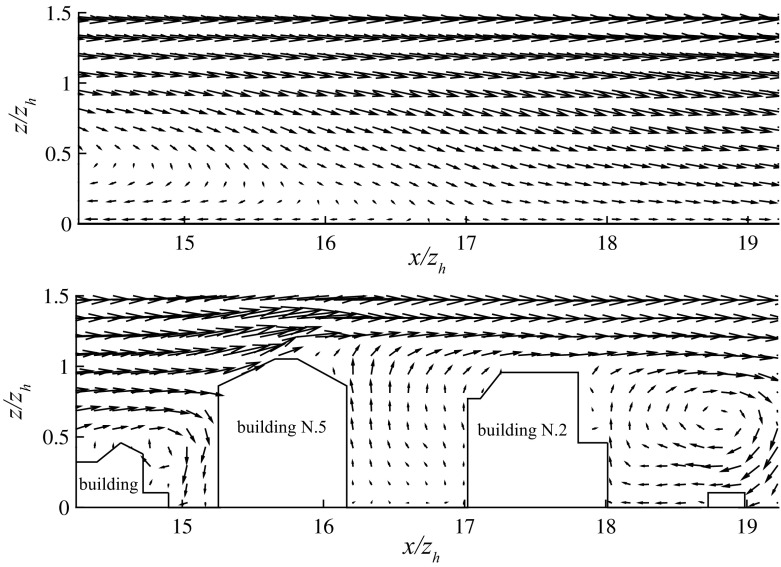
Vector plots of the time-averaged velocity field in the $y^* = 16.73$ plane (passing through the tower location) for simulation A (*top*), corresponding to flow in the along-canyon direction ($\alpha = 66^{\circ }$), and for simulation C (*bottom*), corresponding to an across-canyon wind direction ($\alpha = 156^{\circ }$). The profile tower is located at $x^* = 16.73$ in both plots. Buildings are labeled as in Fig. 1. Vectors are generated on a coarser grid ($2 \varDelta$) for the sake of visualization

Figures 4 and 5 compare DA and locally-sampled (i.e., extracted from the LES at the tower location) time-averaged $\tilde{u}$ and $\tilde{w}$, against the corresponding mean tower-measured data for the two considered approaching wind directions ($\alpha = 66^{\circ }$ and $\alpha = 156^{\circ }$). The locally-sampled time-averaged LES velocity component $\overline{\tilde{u}}^*(x_\mathrm{t}^*,y_\mathrm{t}^*,z^*)$ compares well against $\overline{u}_{\mathrm{tower}}^*$ for both wind directions and all heights. Locally sampled and DA LES results are characterized by a modest standard deviation (shaded regions in the LES profiles) throughout the RSL, underlying the limited influence of both $z_0$ and the SGS closure model in this region of the flow. The relatively larger standard deviation characterizing $\overline{\tilde{u}}^*(x_\mathrm{t}^*,y_\mathrm{t}^*,z^*)$ in the along-canyon wind regime ($\alpha = 66^{\circ }$) is mainly due to variation of the dissipation rates across SGS closures. The component $\overline{\tilde{w}}^*(x_\mathrm{t}^*,y_\mathrm{t}^*,z^*)$ also compares well against the corresponding $\overline{w}_{\mathrm{tower}}^*$ for both along-canyon ($\alpha = 66^{\circ }$) and across-canyon $(\alpha = 156^{\circ })$ wind directions, as displayed in Fig. 5. Flow approaching from $\alpha = 156^{\circ }$ leads to a convergence of the flow in the along-canyon direction, causing a local updraft at the tower location, as apparent in Fig. 6. This behaviour is in agreement with both tower measurements and wind-tunnel results of Feddersen (2005). Further, the lack of a recirculation region for flow approaching from $\alpha = 156^{\circ }$ (across-canyon regime) is consistent Kastner-Klein and Rotach (2004), where street canyons characterized by pitched roofs were connected with no recirculation regions. Flow approaching from $\alpha = 66^{\circ }$ leads to the formation of a long recirculation bubble down wind of building 8 (see Fig. 1), which extends to the tower location (see Fig. 6), hence influencing local statistics. This underlines the strong dependency of the system on the horizontal extension of the computational domain, which should be as large as possible, in particular in the stream wise direction $L_x$, to account for upwind buildings and given the strong correlation of the flow in this coordinate direction. The high variance of $\overline{w}_{\mathrm{tower}}^*$ in Fig. 5 is mainly related to, (a) the small magnitude of mean vertical winds speed, (b) the flow distortion caused by instrument heads in the vertical direction, and (c) the inability to perfectly align sensor heads (no streamline rotation was performed) (Aubinet et al. 2012). DA profiles are characterized by an inflection point $z_{\gamma }$ for both incoming wind directions, suggesting the presence of a mixing-layer type regime, similar to that observed in flow over a uniform strip canopy (Raupach et al. 1991) and in flow over vegetation canopy (Raupach et al. 1996; Hout et al. 2007). Note however that both studies, characterized by roughness of uniform height, identified the inflection point at $z_\mathrm{h}$ (i.e. $z_{\gamma } = z_\mathrm{h}$). In the current study, the inflection point $z_{\gamma }$ coincides with an effective building height $z_e$ (Christen 2005), which can be defined as the averaged surface height, if only buildings higher than $12 \ \mathrm {m}$ are considered. Introducing an effective building height $z_e$ allows description of $z_{\gamma }$ as a function of the surface height distribution, and is justified given that the majority of low buildings in the backyards that make up Mo$_1$ (see Fig. 2) do not influence the flow. Further, relating $z_{\gamma }$ to $z_\mathrm{e}$ allows to recover the limiting behaviour $\lim _{\sigma _{z_\mathrm{h}} \rightarrow 0}{z_{\gamma } = z_\mathrm{e} = z_\mathrm{h}}$ (i.e. when the canopy is characterized by elements of uniform height, the inflection point corresponds to the mean building height). The relatively high location for the inflection point is due to the presence of strong shear layers that separate from the higher roofs and resist penetration by large structures from above (Coceal et al. 2006), thus providing a natural separation layer between high-speed and low-speed regions. Local profiles are very dependent on the specific features of the urban morphology throughout the RSL, and are therefore not representative of DA quantities. For the along-canyon regime ($\alpha = 66^{\circ }$) locally sampled stream wise velocities $(\overline{\tilde{u}}(x_\mathrm{t},y_\mathrm{t},z))$ depart from their DA counterparts $(\langle \overline{\tilde{u}} \rangle )$ in the RSL, mainly due to the persistence of a streamwise elongated low-speed streak, which is locked at the canyon location. This might partly be favoured by the modest vertical and horizontal extensions of the computational domain, which do not allow a full representation of such large-scale structures. However, a similar behaviour was observed in preliminary tests of flow over a larger domain size $(1536 \times 1536 \times 512 \ \mathrm {m})$ (not shown), which suggests that locking of high-speed and low-speed streaks between high-rise buildings is a typical feature of RSL turbulence, and promotes the use of a local scaling approach to collapse profiles in the RSL.

### Momentum Fluxes

Applying the intrinsic DA operator to the LES momentum conservation equation (Eq. 1) results in

6 $$\begin{aligned} \frac{1}{\rho } \frac{\partial \langle \overline{\tilde{p}}_{\infty } \rangle }{\partial x} = - \frac{1}{\lambda _\mathrm{p}(z)} \frac{\partial }{\partial z} \left[ \lambda _\mathrm{p}(z) (\langle \overline{\tilde{u}^{\prime } \tilde{w}^{\prime }} \rangle + \langle \overline{\tilde{u}}^{\prime \prime }\overline{\tilde{w}}^{\prime \prime } \rangle + \langle \overline{\tau }_{xz}^{\mathrm{SGS}} \rangle ) \right] - \frac{1}{\rho } \left\langle \frac{\partial \overline{\tilde{p}}^{\prime \prime }}{\partial x} \right\rangle , \end{aligned}$$

where $\langle \overline{\tilde{u}^{\prime } \tilde{w}^{\prime }} \rangle$ is the DA turbulent momentum flux, $\langle \overline{\tilde{u}}^{\prime \prime }\overline{\tilde{w}}^{\prime \prime } \rangle$ is the so-called dispersive momentum flux, $\langle \overline{\tau }_{xz}^{\mathrm{SGS}} \rangle$ is the SGS contribution to the momentum flux, and $\frac{1}{\rho } \left\langle \frac{\partial \overline{\tilde{p}}^{\prime \prime }}{\partial x} \right\rangle$ is the kinematic pressure drag, which performs work against the imposed pressure gradient from the wall $(z=0)$ up to the height of the tallest building $z_\mathrm{h_\mathrm{max}}$. The layer of air below $z_\mathrm{h_\mathrm{max}}$ is the so-called interfacial layer (Brutsaert 1982). In the considered canopy, buildings occupy a significant fraction of the total volume, thus causing a reduction in the outdoor air volume with depth; this is taken into account through the introduction of the plan-area fraction $\lambda _\mathrm{p}(z)$ parameter in the intrinsic averaging operation, defined as the fraction of space occupied by fluid at a given horizontal plane. Figure 2 displays $\lambda _\mathrm{p}(z)$ for the current set-up. To derive Eq. 6 we have used the averaging theorem (Whitaker 1969), which allows the double averaging of the derivative of a given quantity to be expressed as the derivative of the DA quantity, i.e.

7 $$\begin{aligned} \left\langle \frac{\partial \overline{\theta }}{\partial x_i} \right\rangle = \frac{1}{\lambda _\mathrm{p}(z)} \frac{\partial \lambda _\mathrm{p} (z) \langle \overline{\theta } \rangle }{\partial x_i} - \frac{1}{A_\mathrm{f}}\int _{\partial A_\mathrm{f}} \overline{\theta }(x,y,z) n_i \mathrm{d}l, \end{aligned}$$

where $\theta$ is any non spatially-averaged function, $\mathrm{d}ll$ is an arc element of the curve $\partial A_\mathrm{f}$, and $A_\mathrm{f}$ is a multiply-connected domain, namely the intersection of the constant elevation *z* plane with the solid interface (the buildings). Integrating Eq. 6 analytically in the interval $z \in (z_\mathrm{h_\mathrm{max}},\delta ]$, results in

8 $$\begin{aligned} \frac{1}{\rho } \frac{\partial \langle \overline{\tilde{p}}_{\infty } \rangle }{\partial x}(\delta - z) = \langle \overline{\tilde{u}^{\prime } \tilde{w}^{\prime }} \rangle + \langle \overline{\tilde{u}}^{\prime \prime }\overline{\tilde{w}}^{\prime \prime } \rangle + \langle \overline{\tau }_{xz}^{\mathrm{SGS}} \rangle = \langle \overline{T}_{xz} \rangle (z). \end{aligned}$$

Equations 8 states that the drag that the flow exerts against the imposed pressure gradient varies linearly with height, though this statement does not hold in the interfacial layer, where it is not possible to integrate Eq. 6 analytically. Each term in Eq. 8 scales with $u_{\tau }^2 = (\delta -z_\mathrm{d})\partial _x p_{\infty }/\rho$, hence DA profiles are normalized adopting $u_{\tau }^2$, whereas measured momentum fluxes are first rescaled with $u_{\tau }^2(x_\mathrm{t},y_\mathrm{t},z_\mathrm{t}) / u_{\tau ,\mathrm{tower}}^2(z_\mathrm{t})$, and then also normalized with $u_{\tau }^2$, i.e.

9 $$\begin{aligned} \overline{\tilde{u}_i^{\prime }\tilde{u}_j^{\prime }}_{\mathrm{tower}}^*(z) = \frac{u_{\tau }^2(x_{t},y_{t},z_{t})}{u_{\tau ,\mathrm{tower}}^2(z_{t})} \frac{\overline{\tilde{u}_i^{\prime }\tilde{u}_j^{\prime }}_{\mathrm{tower}}(z)}{u_{\tau }^2}. \end{aligned}$$

#### Turbulent Fluxes

**Fig. 7:**
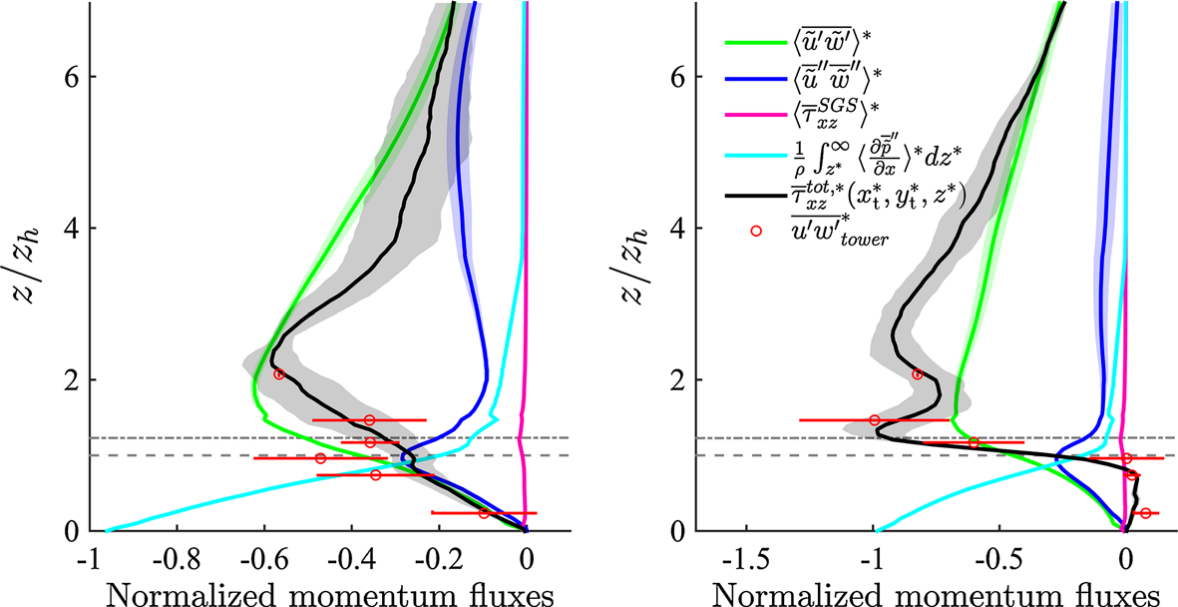
Comparison of vertical kinematic fluxes of streamwise momentum against tower-measured data for the along-canyon (*left*) and across-canyon (*right*) wind directions. *Notation* DA turbulent momentum fluxes $\langle \overline{\tilde{u}^{\prime }\tilde{w}^{\prime }} \rangle ^*$, *green*; dispersive fluxes $\langle \overline{\tilde{u}}^{\prime \prime } \overline{\tilde{w}}^{\prime \prime } \rangle ^*$, *blue*; DA subgrid fluxes $\langle \overline{\tau }_{xz}^{\mathrm{SGS}} \rangle ^*$, *magenta*; DA total pressure drag $\frac{1}{\rho } \int _{0}^{z^*}{\langle \frac{\partial \overline{\tilde{p}}^{\prime \prime }}{\partial x}} \rangle ^* \mathrm{d}z^*$, *cyan*; time-averaged locally-sampled turbulent + SGS momentum fluxes $\overline{\tau }_{xz}^{tot,*}(x_\mathrm{t}^*,y_\mathrm{t}^*,z^*)$, *black*; tower data, *red circles*. *Horizontal dashed* and *dot-dashed* (*grey*) *lines* denote $z_\mathrm{h}$ and $z_{\gamma }$ respectively. Only the lower $75\,\%$ of the domain is shown

**Fig. 8:**
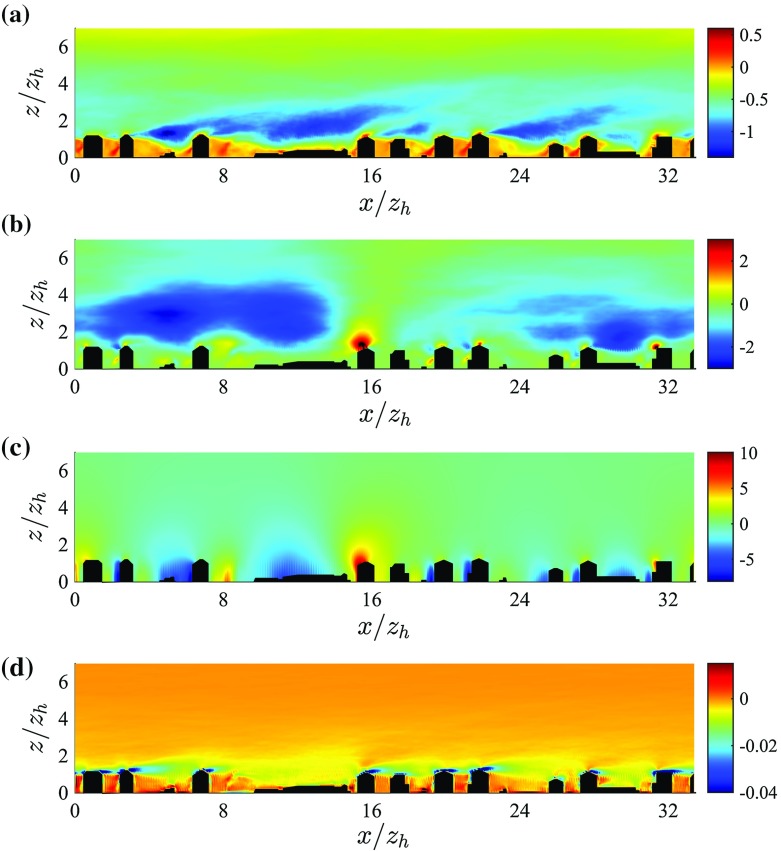
Vertical slices intersecting the tower location (plane $y^*=16.73$) displaying a *colour contour* of turbulent momentum fluxes $\overline{\tilde{u}^{\prime }\tilde{w}^{\prime }}^*$ (**a**), of dispersive momentum fluxes $(\overline{\tilde{u}}^{\prime \prime } \overline{\tilde{w}}^{\prime \prime })^*$ (**b**), of total pressure drag  (**c**) and of SGS fluxes $\overline{\tau }_{xz}^*$ (**d**). Data are from simulation *C* (across-canyon wind direction, $\alpha = 156^{\circ })$. The lower $75\,\%$ of the domain is shown

Measured and numerical turbulent stresses compare well for the across-canyon regime ($\alpha = 156^{\circ }$) whereas LES underpredicts the measured turbulent stress at $z/z_\mathrm{h} \approx 1$ for the along-canyon regime ($\alpha = 66^{\circ }$). Boundary conditions we could not include in the model, such as cars, trees, temporary structures, etc., might contribute to the mismatch. From Fig. 7 it is clear how form drag dominates in the urban canopy layer (UCL)—the layer of air extending from the ground up to the mean height of the buildings, whereas above the UCL the main sink of momentum is from turbulent and dispersive stresses $(\overline{\tilde{u}^{\prime } \tilde{w}^{\prime }}$ and $\langle \overline{\tilde{u}}^{ \prime \prime } \overline{\tilde{w}}^{\prime \prime } \rangle$ respectively). For the across-canyon wind regime it is also apparent how $\langle \overline{\tilde{u}}^{\prime \prime }\overline{\tilde{w}}^{\prime \prime } \rangle \approx \langle \overline{\tau }_{xz}^{\mathrm{SGS}} \rangle \approx 0$ for $z^* \gtrapprox 5$, and thus $\langle \overline{\tilde{u}^{\prime }\tilde{w}^{\prime }} \rangle \approx \frac{1}{\rho } \frac{\partial \langle \overline{\tilde{p}}_{\infty } \rangle }{\partial x}(\delta - z) < 0$. DA turbulent momentum fluxes $\langle \overline{\tilde{u}^{\prime }\tilde{w}^{\prime }} \rangle$ peak above the inflection layer $z_{\gamma }$, presumably due to the advection and turbulent diffusion of wake regions in the (positive) vertical direction, as is apparent from Fig. 8. From Fig. 8 is also clear how the taller buildings play a key role in dictating the properties of turbulent stresses, fixing the length scales of wake turbulence, and sheltering smaller buildings. The spatial distribution of selected terms in Fig. 8 is representative of the entire domain.

#### Dispersive Fluxes

Dispersive fluxes peak at the average building’s height $z_\mathrm{h}$ and are of the same sign and approximate magnitude of their DA turbulent counterpart in the UCL. Results from previous studies focusing on flow over arrays of regular and random surface mounted cubes (Coceal et al. 2006; Martilli and Santiago 2007; Xie et al. 2008; Kono et al. 2010), showed a qualitatively similar trend in the UCL, i.e. dispersive fluxes increase linearly with height up to $z_\mathrm{h}$. However, their magnitude was found to be $0.15 u_{\tau }^2$ at most, likely due to the inherent symmetries characterizing idealized geometries. Dispersive fluxes in flow over gravel beds were also found to be significantly smaller than in the current study, with a maximum of about $0.06 u_{\tau }^2$ (Mignot et al. 2009). As is apparent from Fig. 7, dispersive stresses gradually decrease with height from their peak value (at $z=z_\mathrm{h}$), consistent with results from studies of flow over urban-like obstacles (Xie et al. 2008). The gradual decrease as a function of *z* is justified by the large variance of the surface height distribution $(\sigma _{z_\mathrm{h}} = 0.42 z_\mathrm{h})$. From Fig. 8 it is also clear how dispersive momentum fluxes span a broader range of values when compared against their turbulent counterpart in the RSL, highlighting the strong spatial heterogeneity of such terms and the presence of regions in the UCL where strong contributions to the total momentum flux occur (we were however not able to identify any coherent spatial trend).

#### Pressure Drag

DA total pressure (or form-induced) drag is the main sink of momentum in the UCL. In such a region this drag decreases approximately linearly with height from its surface value $\int _{0}^{\delta }{\frac{1}{\rho } \Big \langle \frac{\partial \overline{\tilde{p}}^{\prime \prime }}{\partial x} \Big \rangle }{\mathrm{d}z} \approx u_{\tau }^2$. The total pressure drag is non-zero up to the height of the tallest building $(z/z_\mathrm{h} =4.18)$, but it is of negligible magnitude above $z/z_\mathrm{h} \approx 1$, when compared against the DA turbulent stresses. As is apparent from Fig. 8, the largest contribution to the form drag arises at the windward side of buildings, where positive horizontal gradients of pressure occur as the flow approaches the facade.

#### Subgrid-Scale Fluxes

SGS fluxes peak at $z_{\gamma } = z_\mathrm{e}$, due to the presence of thin shear layers of fine-scale turbulence (see Fig. 8), but represent a minor contribution to the total momentum flux in the vertical direction. It is important to recall that despite the minor role of SGS terms in the momentum balance, variations in SGS closure, and thus in the related dissipation rates, can have a strong impact on the resolved scale features, via the impact of SGS terms on the kinetic energy of the flow. Given that the wall-modelled stresses are also SGS terms, results suggests that when urban-type surface roughness is directly resolved (through e.g. an immersed boundary method algorithm), the solution is not sensitive to the wall model. This is reassuring, given the lack of a universal law-of-the-wall for flows in complex geometries.

### Budget of TKE

Within the framework of the double averaging, it is possible to expand the total filtered kinetic energy into a temporal and spatial mean (MKE), a wake component (WKE) and a TKE component,

10 $$\begin{aligned} (1/2) \langle \tilde{u}_i \tilde{u}_i \rangle = (1/2) \left( \langle \overline{\tilde{u}}_i \rangle \langle \overline{\tilde{u}}_i \rangle + \langle \overline{\tilde{u}}_i ^{\prime \prime } \overline{\tilde{u}}_i ^{\prime \prime } \rangle + \langle \overline{\tilde{u}_i^{\prime } \tilde{u}_i ^{\prime }} \rangle \right) . \end{aligned}$$

Assuming steady state ($\partial (\cdot )/ \partial t = 0$) and applying first the time averaging $\overline{(\cdot )}$ and subsequently the intrinsic spatial averaging ($\langle \cdot \rangle$) (Nikora et al. 2007; Mignot et al. 2008) results in the DA TKE budget equation,

11 $$\begin{aligned} \frac{1}{2} \frac{ \partial \langle \overline{\tilde{u}_i^{\prime }\tilde{u}_i^{\prime }} \rangle }{\partial t}&= - \langle \overline{\tilde{u}_i^{\prime }\tilde{w}^{\prime }} \rangle \frac{\partial \langle \overline{\tilde{u}}_i \rangle }{\partial z} - \left\langle \overline{\tilde{u}_i^{\prime }\tilde{u}_j^{\prime }}^{\prime \prime } \frac{\partial \overline{\tilde{u}}_i^{\prime \prime }}{\partial x_j} \right\rangle - \langle \overline{\tilde{u}_i^{\prime }\tilde{w}^{\prime }} \rangle \left\langle \frac{\partial \overline{\tilde{u}}_i^{\prime \prime }}{\partial z} \right\rangle \nonumber \\&\quad - \frac{1}{\lambda _\mathrm{p}} \frac{\partial }{\partial z} \left( \lambda _\mathrm{p}(z) \left[ \frac{1}{2}\left\langle \overline{\tilde{u}_i^{\prime }\tilde{u}_i^{\prime }\tilde{w}^{\prime }} \right\rangle + \frac{1}{2} \langle \overline{\tilde{w}}^{\prime \prime } \overline{\tilde{u}_i^{\prime }\tilde{u}_i^{\prime }}^{\prime \prime } \rangle + \langle \overline{\tilde{\pi }^{\prime }\tilde{w}^{\prime }} \rangle \right] \right) \nonumber \\&\quad - \frac{1}{\lambda _\mathrm{p}} \frac{\partial \lambda _\mathrm{p}(z) \langle \overline{\tilde{u}_i^{\prime } \tau _{i3}^{\prime \mathrm{SGS}}} \rangle }{\partial z } + \langle \overline{\tau _{ij}^{\prime \mathrm{SGS}} \tilde{S}_{ij}^{\prime } } \rangle , \end{aligned}$$

where DA shear production $\langle P_\mathrm{s} \rangle = - \langle \overline{\tilde{u}_i^{\prime } \tilde{w}^{\prime }}\rangle \frac{\partial \langle \overline{\tilde{u}}_i \rangle }{\partial z}$, wake production $\langle P_\mathrm{w} \rangle = - \left\langle \overline{\tilde{u}_i^{\prime }\tilde{u}_j^{\prime }}^{\prime \prime }\frac{\partial \overline{\tilde{u}}_i^{\prime \prime }}{\partial x_j} \right\rangle$, work of the time-averaged velocity spatial fluctuations against the DA shear stress $\langle P_\mathrm{m} \rangle = - \langle \overline{\tilde{u}_i^{\prime }\tilde{w}^{\prime }} \rangle \left\langle \frac{\partial \overline{\tilde{u}}_i^{\prime \prime }}{\partial z} \right\rangle$, turbulent transport $\langle T_\mathrm{t} \rangle = - \frac{1}{2 \lambda _\mathrm{p}} \frac{\partial \lambda _\mathrm{p} \left\langle \overline{\tilde{u}_i^{\prime }\tilde{u}_i^{\prime }\tilde{w}^{\prime }} \right\rangle }{\partial z}$, transport by dispersive fluxes $\langle T_\mathrm{d} \rangle = - \frac{1}{2 \lambda _\mathrm{p}} \frac{\partial \lambda _\mathrm{p} \langle \overline{\tilde{w}}^{\prime \prime } \overline{\tilde{u}_i^{\prime }\tilde{u}_i^{\prime }}^{\prime \prime } \rangle }{\partial z}$, pressure transport $\langle T_\mathrm{p} \rangle = - \frac{1}{\lambda _\mathrm{p}} \frac{ \partial \lambda _\mathrm{p} \langle \overline{\tilde{\pi }^{\prime }\tilde{w}^{\prime }} \rangle }{\partial z}$, subgrid transport $\langle D \rangle = -\frac{1}{\lambda _\mathrm{p}} \frac{\partial \lambda _\mathrm{p} \langle \overline{\tilde{u}_i^{\prime } \tau _{i3}^{\prime \mathrm{SGS}}} \rangle }{\partial z }$ and subgrid dissipation $\langle - \epsilon \rangle = \langle \overline{\tau _{ij}^{\prime \mathrm{SGS}} \tilde{S}_{ij}^{\prime } } \rangle$. Given that $\lambda _\mathrm{p}$ varies with height, $\langle P_\mathrm{m} \rangle \ne 0$ (Mignot et al. 2008), and must be accounted for in the TKE budget.

In the current settings MKE is fed into the system through the imposed pressure gradient, and is then partly transformed into TKE through the classic cascade process, and to WKE at scale $z_\mathrm{h}$ due to the work of the imposed pressure gradient against surface drag. Form drag is a sink term for the MKE, but it also subtracts energy from the large shear-generated eddies, short circuiting the normal eddy-cascade process and enhancing the dissipation rate (Raupach and Thom 1981). In the following, the vertical structure of TKE and WKE is first described, to then focus on the TKE budget terms for the two considered wind directions. TKE and WKE scale with $u_{\tau }^2$ and are therefore normalized as previously proposed for momentum fluxes. DA budget profiles are normalized with $u_{\tau } = \sqrt{(\delta -z_\mathrm{d})\partial _x p_{\infty }/\rho }$ and $z_\mathrm{h}$ (e.g. $P_\mathrm{s}^* = P_\mathrm{s} \frac{z_\mathrm{h}}{u_{\tau }^3}$) whereas measured second-order statistics are first rescaled with $u_{\tau }^3(x_\mathrm{t},y_\mathrm{t},z_\mathrm{t}) / u_{\tau ,\mathrm{tower}}^3(z_\mathrm{t})$, and then also normalized with $u_{\tau }$ and $z_\mathrm{h}$, e.g.

12 $$\begin{aligned} P_{\mathrm{s,tower}}^*(z) = \frac{u_{\tau }^3(x_{t},y_{t},z_{t})}{u_{\tau ,\mathrm{tower}}^3(z_{t})} P_{\mathrm{s,tower}}(z) \frac{z_\mathrm{h}}{u_{\tau }^3}. \end{aligned}$$

**Fig. 9:**
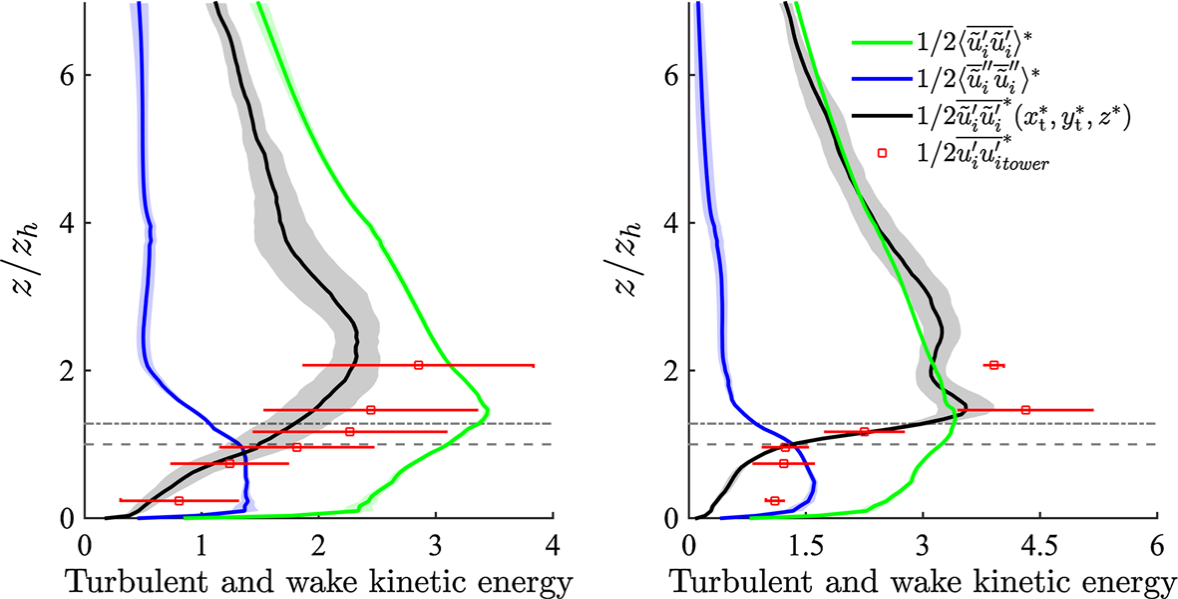
Comparison of turbulent kinetic energy (TKE) and wake kinetic energy (WKE) against tower-measured data for the along-canyon (*left*) and across-canyon (*right*) wind directions. *Notation* DA TKE $1/2 \langle \overline{\tilde{u}_i^{\prime }\tilde{u}_i^{\prime } } \rangle ^*$, *green*; dispersive TKE $1/2 \langle \overline{\tilde{u}}_i^{\prime \prime } \overline{\tilde{u}}_i^{\prime \prime } \rangle ^*$, *blue*; time-averaged locally-sampled TKE $1/2 \overline{\tilde{u}_i^{\prime }\tilde{u}_i^{\prime }}^*(x_\mathrm{t}^*,y_\mathrm{t}^*,z^*)$, *black*; tower data, *red circles*. *Horizontal dashed* and *dot-dashed* (*grey*) *lines* denote $z_\mathrm{h}$ and $z_{\gamma }$ respectively. Only the lower $75\,\%$ of the domain is shown

#### Turbulent and Wake Kinetic Energy

Profiles of TKE and WKE are shown in Fig. 9. Locally sampled time-averaged LES data show relatively good agreement with measurements for the along-canyon wind direction, whereas LES under-predicts the TKE in the UCL and at the location of the highest sonic anemometer for the across-canyon wind regime. This mismatch might be partly due to boundary conditions not included in the model, or to lack of resolution in these delicate regions of the flow. The term $\langle \frac{1}{2} \overline{\tilde{u}_i^{\prime }\tilde{u}_i^{\prime } } \rangle$ peaks at $z_{\gamma }$ for the across-canyon wind regime and slightly above $z_{\gamma }$ in the along-canyon wind regime, to then decrease linearly with height, consistent with tower measurements for the across-canyon wind regime and in agreement with results from flow over random height cubes (Xie et al. 2008). A peculiar feature of the current study is the remarkable magnitude of TKE in the UCL, when compared against results from flow over gravel beds (Mignot et al. 2009) or flow over regular/random arrays of cubes (Coceal et al. 2006; Xie et al. 2008), likely caused by the presence of open areas and organized street canyons. These allow the flow to develop significant MKE, which then cascades into WKE and TKE due to surface drag and the energy cascade process. Further, for both wind directions WKE  $\equiv \langle \frac{1}{2} \overline{\tilde{u}}_i^{\prime \prime } \overline{\tilde{u}}_i^{\prime \prime } \rangle$ is approximately constant within the UCL $(z \le z_\mathrm{h})$ and shows a rapid decay in the lower RSL. The relatively large WKE in the upper RSL for the along-canyon wind regime is again due to locking of streaks in between high-rise structures.

**Fig. 10:**
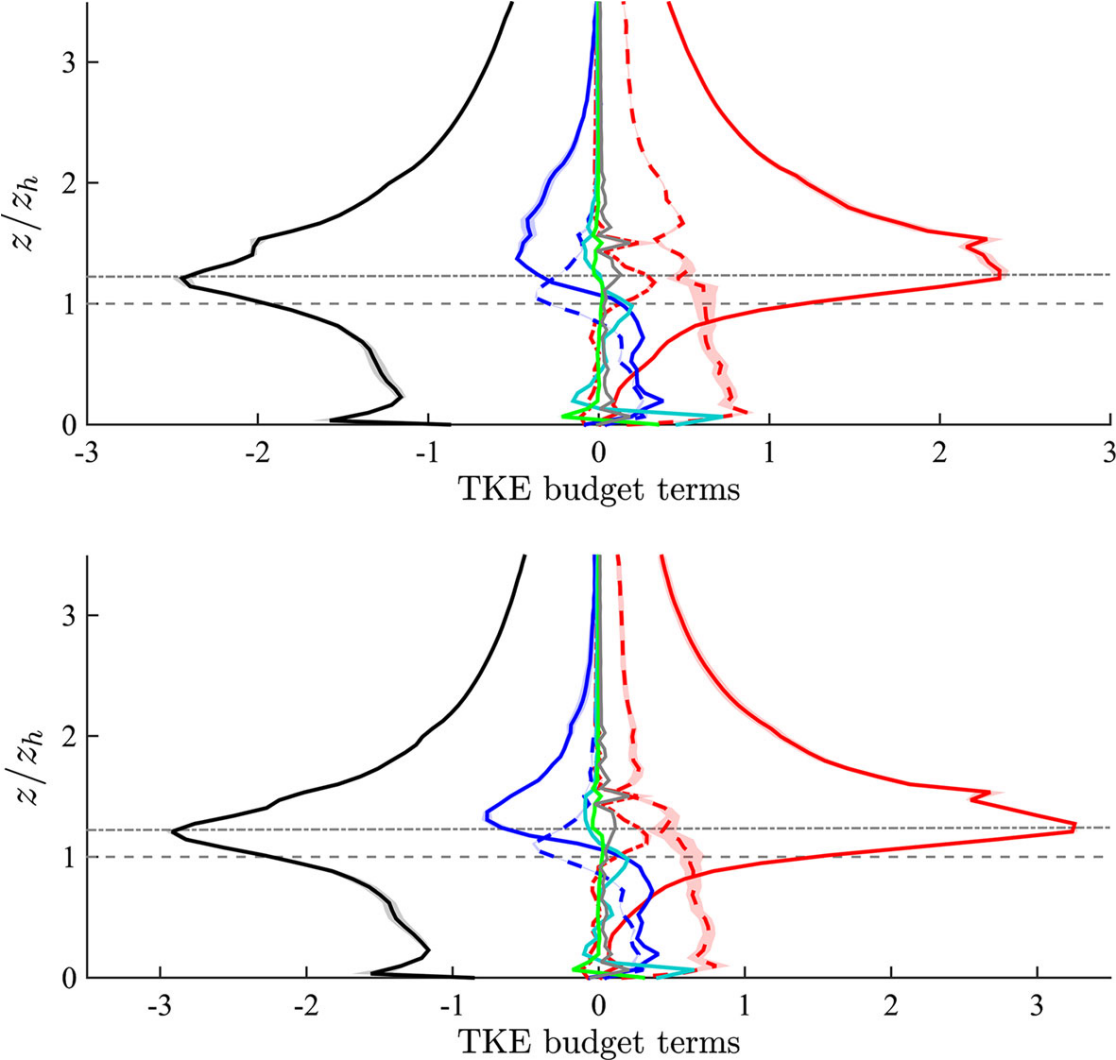
DA TKE budget terms for the along-canyon $\alpha =66^{\circ }$ (*top*) and across-canyon $\alpha =156^{\circ }$ (*bottom*) wind directions. *Notation* turbulent shear production $\langle P_\mathrm{s} \rangle ^*$, *solid red line*; wake production $\langle P_\mathrm{w} \rangle ^*$, *dashed red line*; form-induced production $\langle P_\mathrm{m} \rangle ^*$, *dot-dashed red line*; dissipation $-\langle \epsilon \rangle ^*$, *black*; turbulent transport $\langle T_\mathrm{t} \rangle ^*$, *solid blue line*; dispersive transport $\langle T_\mathrm{d} \rangle ^*$, *dashed blue line*; pressure transport $\langle T_\mathrm{p} \rangle ^*$, *light blue*; subgrid transport $\langle D \rangle ^*$, *green*; residual, *grey*. *Horizontal dashed* and *dot-dashed* (*grey*) *lines* denote $z_\mathrm{h}$ and $z_{\gamma }$ respectively. Only the lower $33\,\%$ of the domain is shown

#### Production Terms

Figure 10 shows that, for both approaching flow angles, DA turbulent shear production $\langle P_\mathrm{s} \rangle$ peaks approximately at the inflection layer $z_{\gamma } = 1.28 z_\mathrm{h}$. This location is connected to thin shear layers that separate from the buildings of near average height, and are advected and diffuse downstream, as displayed in Fig. 13. Previous studies of boundary-layer flow over an uniform strip canopy and of boundary-layer flow over a tree-like canopy also reported a $\langle P_\mathrm{s} \rangle$ peak in correspondence with $z_{\gamma }$ (see Raupach et al. (1991) and Böhm et al. (2013)). $\langle P_\mathrm{s} \rangle$ decreases rapidly from its peak location to approximately zero at the wall, indicating a relatively calm zone in the lower UCL. A second maximum is found in the $\langle P_\mathrm{s} \rangle$ profile at roughly the height of the third mode Mo$_3=22.5 \ \mathrm {m}$ of the *p.d.f.* of building heights (see Fig. 2), which can be regarded as a very specific feature of the current set-up, linked to the shear layers separating from building N. 6 in Fig. 1. $\langle P_\mathrm{w} \rangle$ is the production rate of TKE in the wakes of roughness elements by the interaction of local turbulent stresses and time-averaged strains; in the lower UCL it is approximately constant, positive (WKE converts to TKE) of magnitude $\langle P_\mathrm{w} \rangle ^* \approx u_{\tau }^3/z_\mathrm{h}$. $\langle P_\mathrm{w} \rangle$ accounts for over $50\,\%$ the total production rate of TKE in the UCL, and is therefore non-negligible. A previous study of flow over uniform strip canopy (Raupach et al. 1991) found $\langle P_\mathrm{w} \rangle$ to increase linearly in the canopy, reach a maxima $\langle P_\mathrm{w} \rangle \approx \langle P_\mathrm{s} \rangle$ at $z_\mathrm{h} = z_{\gamma }$, and rapidly decrease to zero in the lower RSL. In experimental and numerical studies of flow over gravel beds (Mignot et al. 2009; Yuan and Piomelli 2014) the magnitude of $\langle P_\mathrm{w} \rangle$ was found to be less than $5\,\%$ of $\langle P_\mathrm{s} \rangle$ (based however on a superficial averaging). $\langle P_\mathrm{w} \rangle$ thus seems to strongly vary as a function of the roughness properties. Our results suggests that in flows over realistic urban canopies the presence of street canyons aligned with the mean flow, open areas and variable building geometries tends to increase $\langle P_\mathrm{w} \rangle$ in the lower UCL ($z^* \lessapprox 0.5$), when compared to results of flow over regular canopy (see for example Raupach et al. (1991)). The additional form-induced production term $\langle P_\mathrm{m} \rangle$ is non-zero only in the vicinity of the inflection layer $z_{\gamma }$, where it accounts for $16\,\%$ the magnitude of $\langle P_\mathrm{s} \rangle$.

**Fig. 11:**
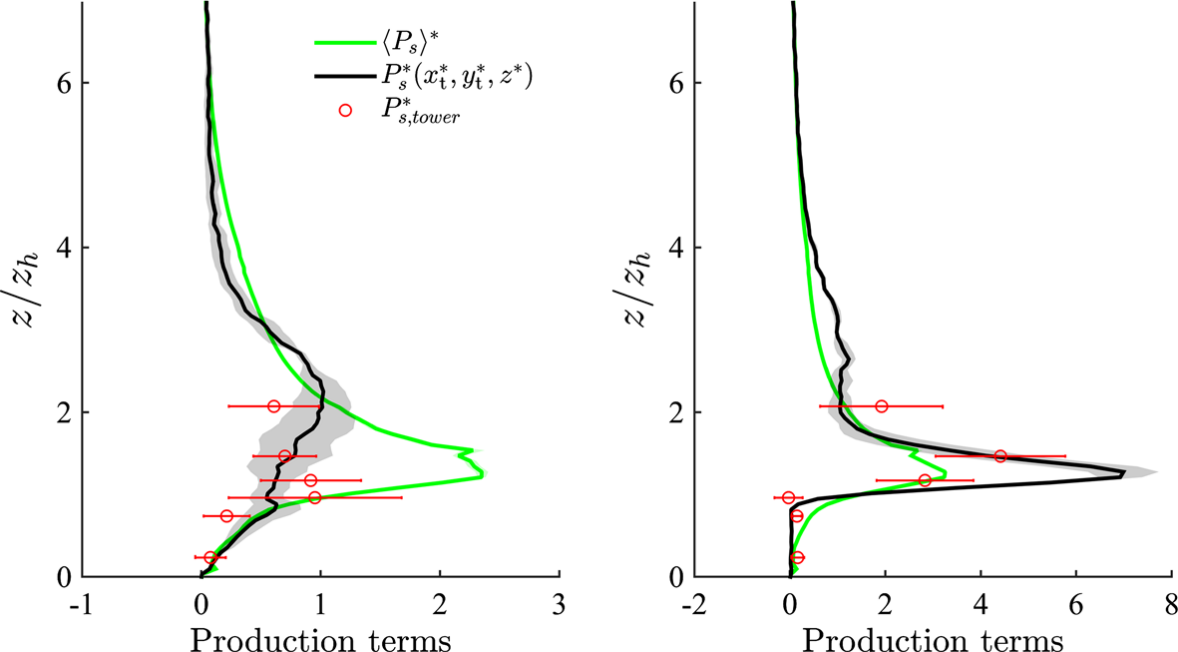
TKE production terms for the along-canyon $\alpha =66^{\circ }$ (*left figure*) and across-canyon $\alpha =156^{\circ }$ (*right figure*) wind directions. *Notation* DA turbulent production $\langle P_\mathrm{s} \rangle ^*$, *green*; locally-sampled time-averaged production $P_\mathrm{s}^*(x_\mathrm{t}^*,y_\mathrm{t}^*,z^*)$, *black*; turbulent production from tower measurements, *red circles*. Only the lower $75\,\%$ of the domain is shown

**Table 3:** Percentage contribution of production, dissipation and transport terms to the total source and sink rate of TKE for the considered layers

Layer	Production	*Dissipation*	Transport
UCL $(0 < z < z_\mathrm{h} )$	$60\,\%\,(+)^*$	$100\,\%\,(-)^*$	$40\,\%\, (+)$
Upper RSL $(z_\mathrm{h} < z < 5 z_\mathrm{h} )$	$100\,\%\, (+)$	$88\,\%\,(-)$	$12\,\%\,(-)$
ISL $(z > 5 z_\mathrm{h} )$	$95\,\%\, (+)$	$100\,\%\,(-)$	$5\,\%\, (+)$

Production ${=} \int _{\mathrm {layer}}{(\langle P_\mathrm{s} \rangle \,{+}\,\langle P_\mathrm{w} \rangle \,{+}\,\langle P_\mathrm{m} \rangle ) }{\, \mathrm{d}z}$, Dissipation ${=} \int _{\mathrm {layer}}{(\langle \epsilon \rangle )}{\, \mathrm{d}z}$, Transport ${=} \int _{\mathrm {layer}}( \langle T_\mathrm{t} \rangle {+} \langle T_\mathrm{d} \rangle \,{+}\, \langle T_\mathrm{p} \rangle \,{+}\, \langle D \rangle ){\, \mathrm{d}z}$

$^{*}\, (+)$ denotes a source of TKE, ($-$) denotes a sink of TKE

Figure 11 compares time-averaged LES profiles, sampled at the tower location, and measured values of shear production. LES results show a remarkable match against measurements, in particular for the across-canyon regime, where the peak in $\langle P_\mathrm{s} \rangle$ is well represented. Based on Fig. 11 locally sampled data prove to be not representative of horizontally-averaged quantities for $\langle P_\mathrm{s} \rangle$. In the across wind regime the tower is located in correspondence of a thin shear layer (see Fig. 13), thus overpredicting the peak in $P_\mathrm{s}$, when compared against its horizontally-averaged counterpart. Conversely, in the along-canyon regime the tower is incapable to properly capture the sharp gradients at $z_{\gamma }$, due to channeling of flow in the “Sperrstrasse” street canyon, which strongly influences local statistics up to the lower RSL regions.

**Fig. 12:**
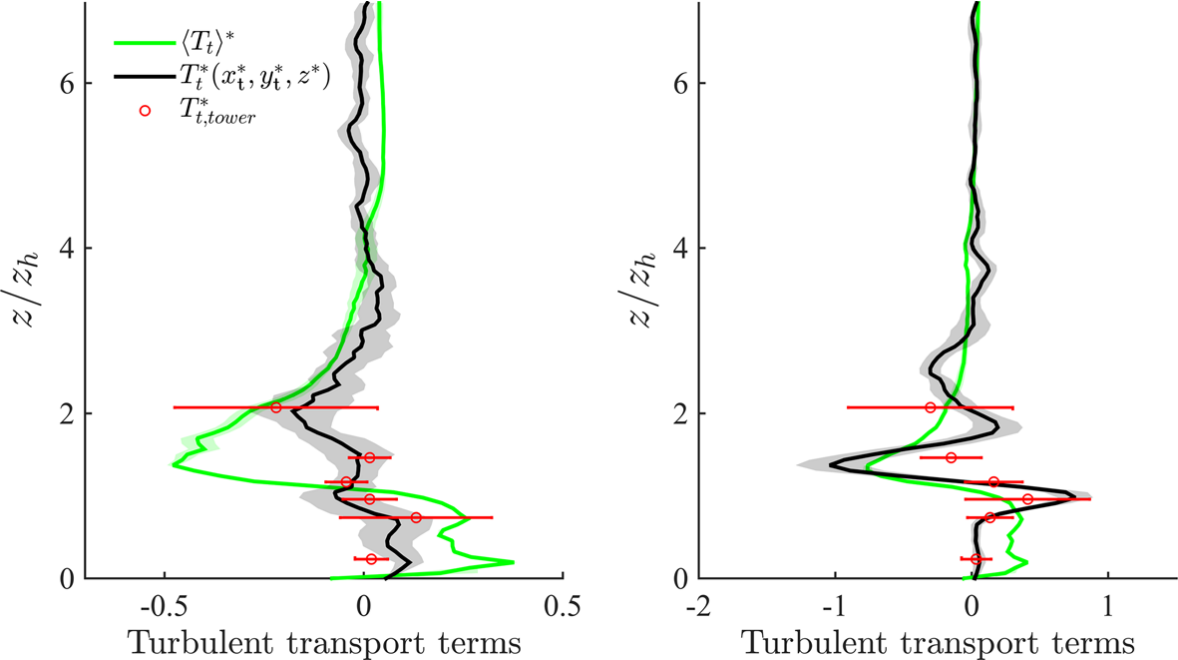
Turbulent transport terms for the along-canyon $\alpha =66^{\circ }$ (*left figure*) and across-canyon $\alpha =156^{\circ }$ (*right figure*) wind directions. *Notation* DA turbulent transport $\langle T_\mathrm{t} \rangle ^*$, *green*; locally-sampled time-averaged turbulent transport $T_\mathrm{t}^*(x_\mathrm{t}^*,y_\mathrm{t}^*,z^*)$, *black*; turbulent transport from tower measurements, *red circles*. Only the lower $75\,\%$ of the domain is shown

#### Transport Terms

**Fig. 13:**
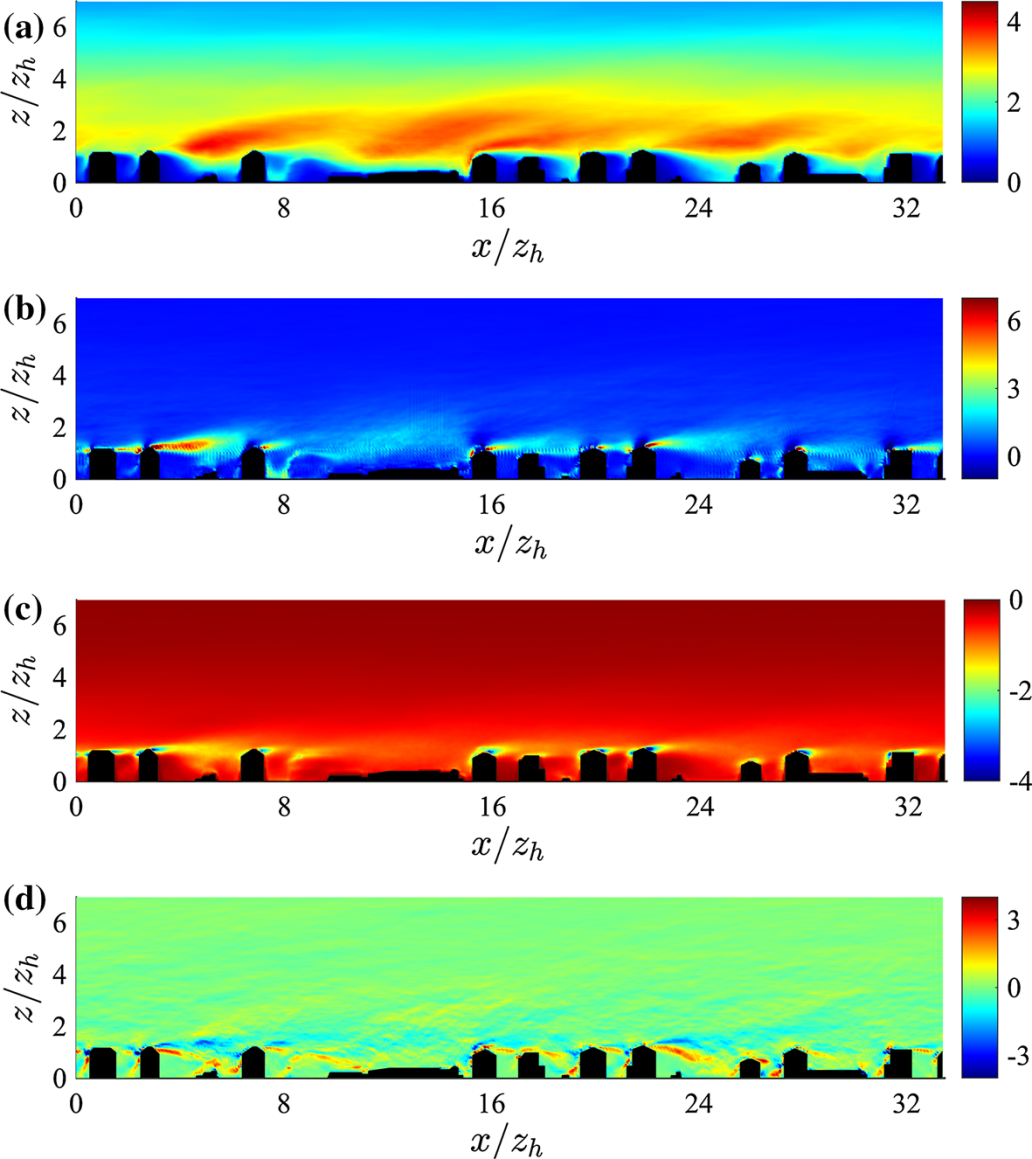
Vertical slices intersecting the tower location (plane $y^*=16.73$) displaying a *colour contour* of TKE$^*$ (**a**), of turbulent shear production $P_\mathrm{s}^*$ (**b**), of dissipation $-\epsilon ^*$ (**c**), and total transport $T_\mathrm{tot}^* = T^*_\mathrm{t} + T_\mathrm{d}^* + T_\mathrm{p}^* + D^*$ (**d**). Data are from simulation *C* (across-canyon wind direction, $\alpha = 156^{\circ })$. The lower $75\,\%$ of the domain is shown

From Fig. 10 it is apparent how DA production terms $(\langle P_\mathrm{s} \rangle + \langle P_\mathrm{w} \rangle + \langle P_\mathrm{m} \rangle )$ overcome dissipation in the RSL down to $z_\mathrm{h}$, i.e. $z_\mathrm{h} \le z \le 5 z_\mathrm{h}$, and DA transport terms are responsible to remove TKE from this layer of high production, and transport it towards the wall to balance dissipation. In the upper RSL $(z_\mathrm{h} < z < 5 z_\mathrm{h})$ transport terms are thus negative, and contribute to about $12\,\%$ the total sink rate of TKE (see Table 3). They change sign in the UCL, where they are of highest significance, contributing to about $40\,\%$ the total source rate of TKE (see Table 3). $\langle T_\mathrm{d} \rangle$ appears as a modulation of $\langle T_\mathrm{t} \rangle$, whereas $\langle T_\mathrm{p} \rangle$ is significant at $z_{\gamma }$ (where it is a sink of TKE) and in the very near wall regions, where it peaks at $\langle T_\mathrm{p} \rangle ^* = 0.8 u_{\tau }^3/z_\mathrm{h}$. Our profiles are in agreement with results of flow over vegetation canopy and with results of flow over gravel beds for the $\langle T_\mathrm{p} \rangle$ term, i.e. turbulence in the lowest levels of a canopy is partly induced by pressure perturbations (Shaw and Zhang 1992; Yuan and Piomelli 2014). An additional spatial characterization of transport terms is provided in Fig. 13. As apparent, transport terms peak at the boundaries of the thin shear layers that separate from the top of the buildings, further justifying the observed DA one-dimensional profiles. Furthermore, the modest standard deviation of DA $\langle T_\mathrm{t} \rangle$ terms for both approaching wind directions confirms once again the insensitivity of the solution with respect to variations in the SGS model and $z_0$ parameter, when the (urban) roughness is explicitly resolved.

Turbulent transport terms are compared against tower measurements in Fig. 12. Numerical results and measurements are in good agreement, apart from an overshoot of the numerical $T_\mathrm{t}(x_{t},y_{t},z)$ in the across-canyon regime at the height of sonic *E*, suggesting higher resolution might be necessary in order to properly describe the small scale turbulence characterizing the thin shear layers that separate from the roofs of buildings (recall that the current grid stencil is 1 m). Note that in this specific case, DA profiles are in qualitative agreement with data from the same tower, and an additional tower (not shown), operated under a much wider range of stabilities during BUBBLE (Christen et al. 2009).

#### Dissipation and Residual Terms

DA dissipation $\langle -\epsilon \rangle$ peaks at $z_{\gamma }$, as displayed in Fig. 10. This is another peculiar feature of the current study, and is in contrast with results of flow over gravel beds (Mignot et al. 2009; Yuan and Piomelli 2014), where the peak in dissipation was found to be shifted toward the wall, with respect to the peak in the shear production rate. Further, a strong rate of dissipation characterizes the very near-wall regions. This peak is required in order to balance pressure transport of TKE from aloft, again confirming the important role of pressure correlation terms in the vicinity of the wall, in flows over directly resolved building interfaces. Figure 13 underlines how the local dissipation rate spatially resembles the local shear production rate, being significant in the shear layers that separate from the buildings. From Fig. 13 it is also apparent how dissipation is significant in the vicinity of the facades of buildings, locally balancing transport terms. The relatively modest residual (see Fig. 10) in the computed TKE budget further validates the numerical results. The finite residual is likely due to spatial interpolation of variables in the near interface regions (required to compute certain TKE budget terms), which leads to numerical truncation errors affecting the quality of computed terms.

**Table 4:** Normalized horizontal standard deviation ($\sigma ^*$) for selected statistics

Quantity	$0 \le z/z_\mathrm{h} < 0.5$	$0.5 \le z/z_\mathrm{h} < 1$	$1 \le z/z_\mathrm{h} < 3$	$3 \le z/z_\mathrm{h} < 5$
$\sigma ^*_\mathrm{TKE}$	0.6	0.5	0.25	0.2
$\sigma ^*_{u^{\prime }w^{\prime }}$	2.3	1.2	0.45	0.35
$\sigma ^*_{P_\mathrm{s}}$	70	10	5	6
$\sigma ^*_{T_\mathrm{t}}$	20	43	37	120
$\sigma ^*_{-\epsilon }$	4	3.5	4	4

Results have been averaged over all the runs and over the considered $z/z_\mathrm{h}$ interval. $\sigma ^* \equiv \sigma (\theta )/\langle \theta \rangle$, where $\theta (x,y,z)$ is a given statistic, $\sigma (\theta ) = \sqrt{ (1/N) \sum (\theta -\langle \theta \rangle )^2}$, and *N* denotes the number of collocation nodes in a horizontal layer ($N_x \times N_y$)

### On the Representativeness of Local Measurements in the RSL

As stated in Sect. 1, field-studies are usually sampling the flow at few points in space, and therefore cannot account for its spatial variability and for dispersive contributions. The very nature of RSL turbulence hence questions the usage of point measurements as surrogate of horizontally averaged quantities in such regions, as underlined in Rotach (1993a, b) and Christen et al. (2009). Unfortunately, the vast range of urban geometries limits the scope of any investigation aiming at defining confidence bounds for locally measured quantities. Without ascribing generality to the proposed results, we here summarize the spatial variability of turbulent statistics and the contribution of dispersive terms in the RSL for the considered study. Such information is of use to ensure the representativeness of local measurements on sites.

Table 4 provides reference values for the normalized horizontal standard deviation $\sigma ^*$ of selected (measurable) flow statistics, averaged over the considered *z* intervals. $\sigma ^*$ is related to sampling at different horizontal locations in space (within the fluid only) and is defined as

13 $$\begin{aligned} \sigma ^*(z) \equiv | \sigma (\theta (z))/\langle \theta \rangle (z) |, \end{aligned}$$

where $\theta$ is a generic flow statistic, $\sigma (\theta ) = \sqrt{ (1/N) \sum (\theta -\langle \theta \rangle )^2}$, and *N* denotes the number of collocation nodes in a horizontal layer considering fluid areas only (i.e. not within buildings). Quantities $\sigma _\mathrm{TKE}^*$ and $\sigma _{u^{\prime }w^{\prime }}$ are characterized by a monotonic decrease from their surface value, but remain finite throughout the UCL and RSL. Based on current results, local measurements of TKE and $u^{\prime }w^{\prime }$ should account for a standard deviation up to about 60 and $230\,\%$ the magnitude of the corresponding sampled mean in the (lower) UCL. The same values decrease to 25 and $45\,\%$ respectively in the above-UCL regions $(z > z_\mathrm{h})$. Note that the proposed percentages are in qualitative agreement with results displayed in Figs. 7 and 9. Table 4 highlights a remarkable spatial variability of $P_\mathrm{s}$ and $T_\mathrm{t}$ the RSL, tightly related to the strength of shear layers that characterized the flow in such regions (as apparent from Fig. 13). Sensor deployment within the RSL should therefore be performed avoiding such high shear rate regions, which would otherwise cause an overestimation of the measured $P_\mathrm{s}$ and $T_\mathrm{t}$, relative to their spatial mean. This is confirmed by results in Figs. 11 and 12: in the across-canyon wind regime ($\alpha =156^{\circ }$) sonics *C*, *D*, *E* (see Table 1) are sampling within one of such shear layers, and the resulting values of $P_\mathrm{s}$ and $T_\mathrm{t}$ are clearly not representative of their spatially averaged value. Note that the large $\sigma ^*_{T_\mathrm{t}}$ in the upper RSL ($3 \le z/z_\mathrm{h} < 5$) is likely related to the negligible magnitude of $T_\mathrm{t}$ in such layer. Besides, the magnitude of the computed coefficients in the RSL is likely amplified by the presence of a relatively taller building (building N.7 in Fig. 1), whose effects on the resulting $\sigma ^*$ remain significant up to a height of $z/z_\mathrm{h} \approx 5$.

**Table 5:** Ratio of dispersive to Reynolds contributions ($\xi$) for selected statistics

Quantity	$0 \le z/z_\mathrm{h} < 0.5$	$0.5 \le z/z_\mathrm{h} < 1$	$1 \le z/z_\mathrm{h} < 3$	$3 \le z/z_\mathrm{h} < 5$
$\xi _\mathrm{TKE}$	1.3	0.9	0.3	0.2
$\xi _{u^{\prime }w^{\prime }}$	0.6	0.8	0.2	0.3
$\xi _{P_\mathrm{s}}$	14	1.5	0.3	0.4
$\xi _{T_\mathrm{t}}$	0.9	0.9	0.5	0.5

Results have been averaged over all the runs and over the considered $z/z_\mathrm{h}$ interval. $\xi \equiv \theta ^\mathrm{d}/\theta ^\mathrm{R}$, where $\theta ^\mathrm{d}$ is the dispersive component of a considered quantity, and $\theta ^\mathrm{R}$ is its Reynolds counterpart

The previous sections have shown that dispersive contributions to the TKE, to the total vertical momentum flux, and to the TKE budget can be significant in the RSL. Table 5 summarizes the relative importance of dispersive terms for different layers. The parameter $\xi$ is introduced, defined as the ratio of dispersive-to-Reynolds contribution for a given quantity,

14 $$\begin{aligned} \xi \equiv | \theta ^\mathrm{d}/\theta ^\mathrm{R}|, \end{aligned}$$

where $\theta ^\mathrm{d}$ is the dispersive component of a considered flow statistic, and $\theta ^\mathrm{R}$ is its Reynolds counterpart. As apparent, dispersive terms are of the same order of magnitude of their corresponding Reynolds component in the UCL for most of the considered quantities, and are also non-negligible in the RSL. Worth noting is that $\xi _{P_\mathrm{s}} = \mathcal {O}(10)$ for $0 \le z/z_\mathrm{h} < 0.5$, which, considering that $\sigma _{P_\mathrm{s}}^* = 70$, suggests point-wise measurements of $P_\mathrm{s}$ in the lower UCL are flawed.

Overall, Tables 4 and 5 suggest that point-wise measurements of TKE and $u^{\prime }w^{\prime }$ are equally biased by the spatial heterogeneity of the flow statistics and by the presence of additional dispersive contribution from the mean flow. Conversely, local sampling of TKE budget terms is largely biased by their spatial heterogeneity, which despite the remarkable magnitude of the $\sigma ^*$ parameters, does not lead to significant contributions from the mean flow (exemplified by the relatively modest $\xi$ values).

## Conclusions

A characterization of mean flow and turbulence in the RSL of a realistic urban canopy, representing a subset of the city of Basel in Switzerland, has been performed via a series of large-eddy simulations (LES) and results have been compared to direct tower measurements from a long-term field campaign. First-order and higher order statistics compare well against tower measurements, confirming that LES in conjunction with the immersed boundary method is a valuable tool for the simulation of flow and dispersion over realistic urban surfaces. Double averaged numerical profiles are not sensitive to variations in both the subgrid-scale (SGS) model and the hydrodynamic roughness length $(z_0)$ parameter, given that form drag represents a significant percentage of the total surface drag, and is well resolved through the IBM. Double-averaged velocity profiles are characterized by an inflection point $z_{\gamma }$, located above the mean building height $z_\mathrm{h}$, highlighting the presence of a mixing-layer type flow regime. Double-averaged Reynolds fluxes and double averaged turbulent kinetic energy (TKE) peak above $z_{\gamma }$, in agreement with results from studies of flow over simplified urban-like surfaces. TKE is significant in the urban canopy layer (UCL), when compared against results of flow over gravel beds and over regular / random arrays of cubes, mainly due to the presence of flow-aligned street canyons, open areas and a variable building height, which strongly increase the strength of both mean kinetic energy and TKE in such regions. Further, dispersive momentum fluxes and dispersive production and transport of TKE are found to be non-negligible in the UCL, and of the same order of magnitude of their Reynolds counterparts. TKE is primarily produced at $z_{\gamma }$ by shear, and is transported down into the cavities of the urban canopy (street canyons, backyards) by turbulent and dispersive transport terms, which share similar magnitudes. Transport terms are non-negligible throughout the RSL. They are of negative sign and contribute to about $12\,\%$ the total variation rate of TKE in the upper RSL ($z_\mathrm{h} < z < 5 z_\mathrm{h}$), whereas they are of highest significance in the UCL ($0 < z < z_\mathrm{h}$), where they are of positive sign and contribute to about $40\,\%$ the local variation rate of TKE. Wake production is roughly constant up to $z_{\gamma }$ and of non-negligible magnitude $(\langle P_\mathrm{w} \rangle ^* \approx u_{\tau }^3/z_\mathrm{h})$, contributing up to $50\,\%$ the total TKE production rate in the UCL. Further, pressure transport is found to be a significant source of TKE in the near-wall regions, in agreement with previous findings of flow over vegetation canopy and flow over gravel beds. The spatial heterogeneity and the dispersive contribution of selected flow quantities are summarized for reference intervals in the RSL. Results highlight how RSL tower measurements can be severely biased because of the spatial heterogeneity of the flow. Further, tower measurements cannot be used to quantify all terms in a horizontally-averaged view: dispersive terms are important in a real canopy. This also means that one-dimensional exchange models in urban canopy parametrizations relying commonly solely on turbulent fluxes will underestimate the exchange. Dispersive fluxes should therefore be considered in the exchange computation of future urban canopy parametrization schemes.

## Electronic supplementary material

Below is the link to the electronic supplementary material.

Supplementary material 1 (mp4 5118 KB)

Supplementary material 2 (mp4 3341 KB)
